# Spatiotemporal assessment of urban PM₁₀ and population exposure using integrated satellite, meteorological, and socio-spatial data

**DOI:** 10.3389/fpubh.2026.1940025

**Published:** 2026-08-31

**Authors:** Tudor Caciora, Grigore Vasile Herman, Monica Costea, Vasile Grama, Thowayeb H. Hassan, Tomasz Oberski

**Affiliations:** 1 Department of Geography, Tourism and Territorial Planning, Faculty of Geography, Tourism and Sport, University of Oradea, Oradea, Romania; 2 Faculty of Environmental Protection, University of Oradea, Oradea, Romania; 3 Social Studies Department, College of Arts, King Faisal University, Al Ahsa, Saudi Arabia; 4 Department of Geodesy and Geoinformatics, Faculty of Civil Engineering, Environmental and Geodetic Sciences, Koszalin University of Technology, Koszalin, Poland

**Keywords:** aerosol optical depth, exposure index, human health risk, PM₁₀ exposure, satellite-based air quality assessment, socio-spatial indicators, urban air pollution

## Abstract

**Introduction:**

The pollution with particulate matter is a major risk factor for population health, especially in urban environments, where the interaction between anthropogenic sources, meteorological conditions and built environment characteristics generates complex spatial patterns of exposure. In this context, the present study aims to estimate the spatial distribution of PM₁₀ concentrations and assess population exposure in a medium-sized city in Romania by integrating satellite data, *in situ* measurements and socio-spatial indicators.

**Methods:**

The methodology is based on MODIS-MAIAC satellite products to retrieve Aerosol Optical Depth (AOD) values, which are correlated with PM₁₀ data and meteorological variables from four ground-based air quality monitoring stations. Based on these data, a multiple regression model was developed to estimate PM₁₀ concentrations, and subsequently, a composite exposure index was constructed by integrating relevant socio-spatial indicators, such as population density, built environment characteristics, the Normalized Difference Vegetation Index (NDVI), and terrain elevation.

**Results and discussion:**

The results highlight pronounced seasonal variability in PM₁₀ concentrations, with maximum values in the cold season (up to 41.8 μg/m^3^) and an annual average of approximately 20.5 μg/m^3^, exceeding the thresholds recommended by the World Health Organisation but falling within the limits set by the European Union. The direct relationship between AOD and PM₁₀ is relatively weak (*R* = 0.28), but integrating meteorological variables significantly improves the model’s performance. The analysis of the composite index indicates that approximately 20.1% of the population of the Oradea Metropolitan Area lives in areas with a high predisposition to exposure, particularly near industrial areas and in densely built urban environments. The results confirm that population exposure to PM₁₀ is determined not exclusively by concentration levels but by the complex interactions among pollution, population distribution, and the characteristics of the built environment. The proposed integrated approach provides a robust tool for identifying vulnerable areas and supporting urban planning and air quality management, contributing to the development of strategies to reduce risks to human health.

## Introduction

The negative effects of particulate matter (PM) on human health are well documented and are associated with respiratory and cardiovascular diseases, such as asthma, emphysema, and lung cancer (1–3). Globally, air pollution is responsible for millions of premature deaths annually, with estimates ranging from over 4 million (4) to approximately 6.7 million, according to the World Health Organisation (WHO), with PM being among the main pollutants responsible for these (5). In this context, reducing PM concentrations, especially in urban environments, is a major priority, but the processes underlying their accumulation are highly complex, influenced by dynamic interactions among emission sources, meteorological conditions, and the particularities of the built environment (6). These processes are not yet fully understood and exhibit significant variation across areas, making it difficult to generalize the relationships between determinants and pollution levels (7, 8). Furthermore, while in many European cities air quality monitoring networks are relatively well developed, in other regions they are limited or insufficiently spatially distributed (especially in Eastern Europe), which reduces the representativeness of the data and the ability to adequately capture the processes of PM accumulation and dispersion (9, 10). Even in cases where networks are well represented, their spatial distribution does not allow for precise interpolations and does not provide a complete and coherent overview of the spatial variability of pollution. Besides the identification of particulate matter, satellite-based techniques have been increasingly employed for monitoring a wide range of atmospheric pollutants, including NO₂, SO₂, CO, CH₄, O₃. Recent studies have further demonstrated the potential of Sentinel-5P observations integrated within Google Earth Engine for large-scale air quality monitoring and spatiotemporal analyses of these pollutants, highlighting the advantages of cloud-based geospatial platforms for environmental assessment and air quality management (11–13).

Understanding the processes controlling PM distribution requires extensive and spatially continuous data, but this is not available for much of the world, satellite-based observations of Aerosol Optical Depth (AOD) are frequently used in air quality assessment, as they have the ability to fill gaps generated by the network of ground-based air quality monitoring stations and generate a continuous representation (14–16). The use of AOD as a proxy for ground-level PM concentrations is frequently applied but has several limitations. AOD represents the average of particles over the entire atmospheric column, whereas PM reflects their mass at ground level at a specific point, which can lead to discrepancies between the two variables (17). In addition, the relationships between AOD and PM are strongly influenced by meteorological elements (MEs) and aerosol composition, making them difficult to generalize across regions without local calibration (18). Also, the lack of extensive measurements of the vertical distribution of aerosols limits the ability to generalize these relationships to large scales (19). However, AOD remains a useful indicator for assessing indirect pollution, especially when integrated with additional variables, such as ME, land use, or temporal variables, which improve model performance (20, 21). The integration of these variables enables the capture of seasonal variability and long-term trends in PM concentrations, as well as the identification of potential pollution sources and exposed areas (8).

Based on the above, the present study aims to identify areas with a high predisposition to impact the health of the population within the Oradea Metropolitan Area (OMA). The integration of composite indices for modelling PM_10_ concentrations in 2025 was considered, based on AOD values derived from satellite data, PM10 values and MEs measured *in situ* at air quality monitoring stations, and a set of relevant socio-spatial indicators (SSIs). This study is based on the hypothesis that the spatial distribution of population exposure to PM₁₀ is not determined exclusively by the level of concentrations of this pollutant and the positioning of emission sources, but by the complex interaction between pollution, meteorological conditions and SSIs of the analyzed environment, such as population density and proximity to emission sources, among others. The novelty of the research lies in the development of an integrated analysis framework that combines satellite data, *in situ* measurements and SSIs in a composite susceptibility index, capable of highlighting spatial variations in exposure and identifying vulnerable areas from the perspective of risks to population health. At the same time, the analysis quantified the exposed population by determining the number of inhabitants within the OMA who live in areas characterized by a high predisposition to pollution, thus providing an integrated perspective on the distribution of risk from both spatial and demographic perspectives. In addition, the study targets an area that has not been sufficiently investigated previously from the perspective of using satellite data to analyze atmospheric pollution, specifically the OMA. To date, only the studies by El Kihel et al. (22) and Caciora et al. (23) have addressed this region with regard to satellite, pollution, and MEs data, but they focused exclusively on extracting Land Surface Temperature from Landsat 8 and MODIS data. At the same time, the research focuses on a medium-sized city at the European level, an urban category relatively poorly represented in the specialized literature, although these cities house a significant share of the population and exhibit specific dynamics of air pollution and exposure.

## Materials and methods

### Case study

**Figure 1:**
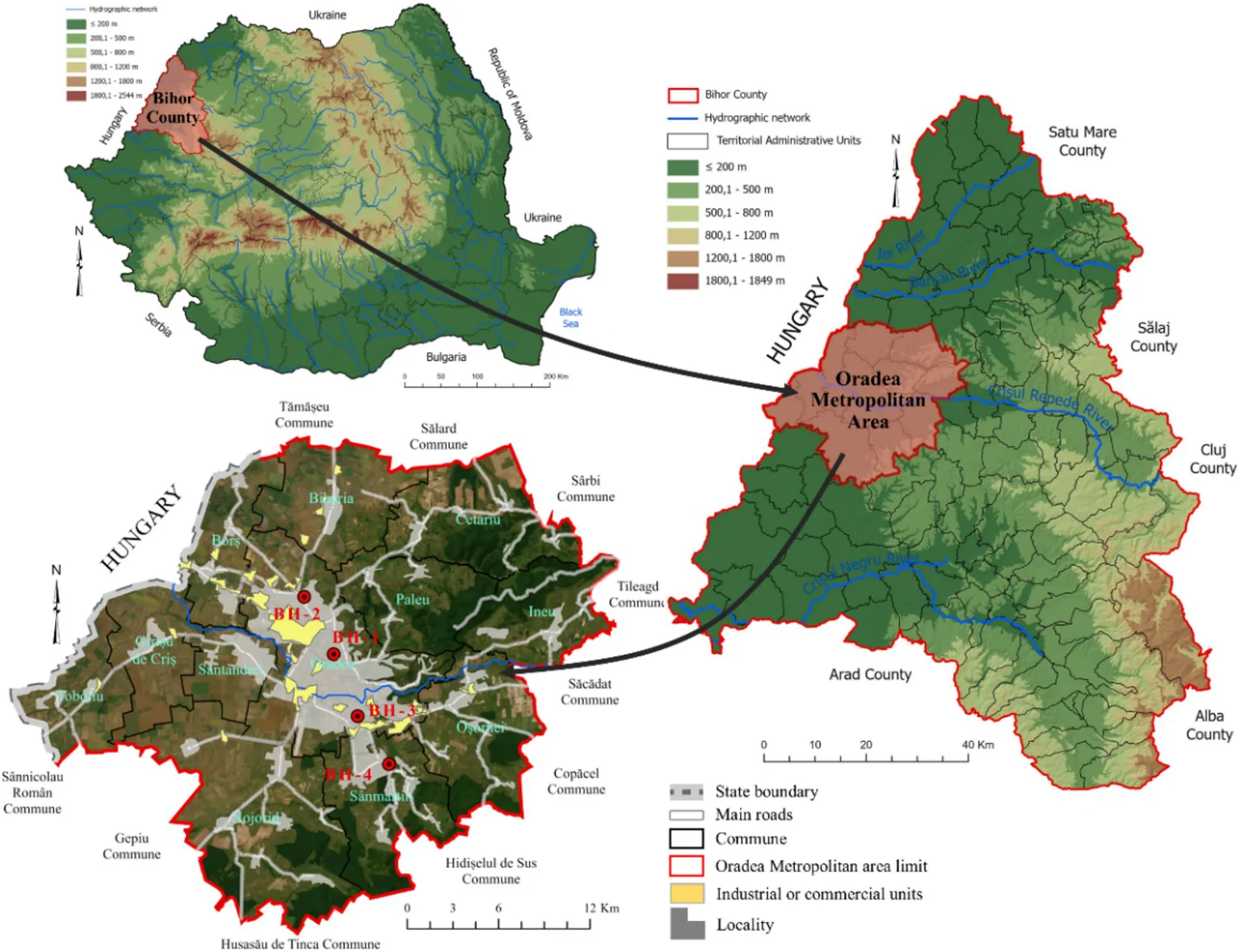
Location of the study area (Oradea Metropolitan Area) within Romania and Bihor County.

The study area for the present scientific approach is the OMA, located in western Bihor County, on the border with Hungary, which constitutes an urban-peri-urban system comprising 13 administrative-territorial units. This includes the Oradea Municipality (212,888 inhabitants in 2025) and a periurban area with 68,094 inhabitants, characterized by a strong urban–rural functional interdependence. The OMA includes 43 localities (1 urban and 42 rural). Altitudes range from 91 to 344 m, with predominantly low relief and slight hilly forms in the northeastern sector (Figure 1).

From a climatic point of view, the region belongs to the temperate-continental zone, with oceanic influences, having an average annual temperature of ~10 °C and precipitation of ~600 mm/year. At ground level, air circulation is dominated by winds from the southern and southwestern sectors, with average speeds of approximately 3 m/s, while in the free atmosphere, westerly winds are individualized (24–26).

The OMA covers approximately 750 km^2^, of which about 120 km^2^ is built-up area, while land use is predominantly agricultural (≈432 km^2^), followed by forest areas (≈120 km^2^), water bodies (≈10 km^2^) and industrial and commercial areas (≈16 km^2^), forming a mixed urban–rural functional landscape (Figure 1). Agricultural activities, industrial areas and heavy road traffic, together with residential heating (especially in the cold season) and particle resuspension, constitute major sources of PM. At the same time, the urban–periurban structure and daily mobility generate complex spatial patterns of population exposure to PM pollution. In this context, assessing the spatial distribution of PM₁₀ at a metropolitan scale is essential for understanding the risks to population health.

In recent decades, the OMA (especially the Oradea Municipality) has experienced rapid urban expansion, with the development of built-up areas particularly evident in the western and southern sectors. These land-use transformations, characterized by the conversion of vegetated or sparsely built-up areas into denser, more impervious urban areas, reflect an active urban dynamic. In this context, the city’s continued expansion may intensify PM₁₀ pollution by increasing anthropogenic sources and altering local conditions for pollutant dispersion. At the same time, in recent years, particularly after Romania’s accession to the European Union, the Oradea Municipality has undergone an industrial restructuring process, characterized by the reduction of highly polluting activities and the shift towards less emission-intensive industries, such as automotive component production, logistics, and the textile industry. In this context, it remains relevant to assess the contribution of these activities to PM levels to determine their potential to generate air pollution.

### Data acquisition and processing

**Figure 2:**
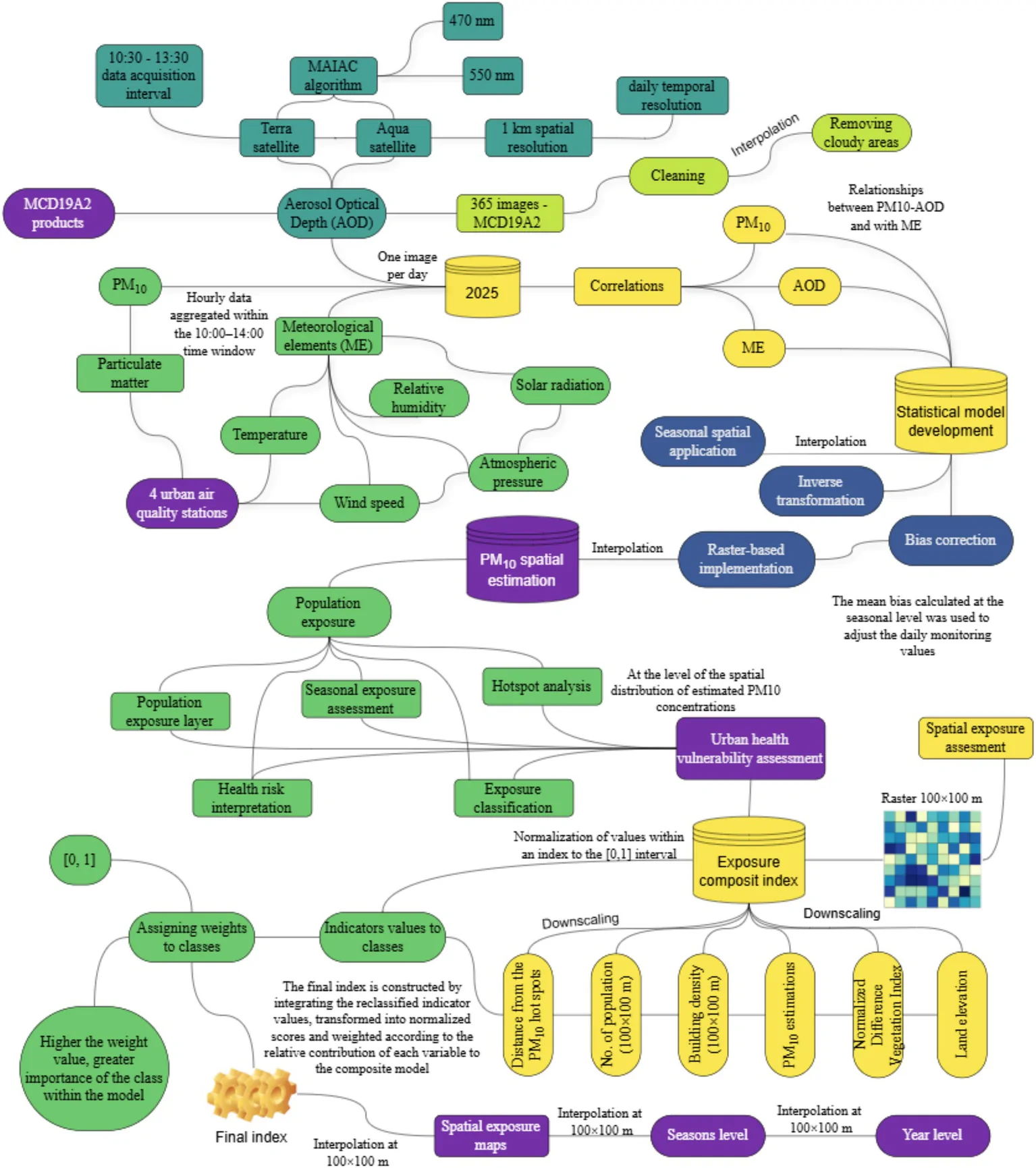
Spatiotemporally generalized MODIS-MAIAC AOD–driven framework for urban PM₁₀ estimation, including meteorological forcing, bias-corrected raster implementation, and the construction of a composite exposure index.

The data acquisition and processing considered three main sources for the year 2025. On the one hand, the Moderate Resolution Imaging Spectroradiometer-Multi-Angle Implementation of Atmospheric Correction (MODIS-MAIAC) (MCD19A2) satellite products were used to obtain AOD values. On the other hand, to estimate PM₁₀ concentrations based on AOD and MEs, data from the four air quality monitoring stations in the OMA were used. In addition, to build the composite predisposition index, SSI data from the Copernicus program, along with variables derived from satellite imagery and other local sources, were used to characterize the built environment and population distribution (Figure 2).

The proposed methodology is transferable and can be applied to other cities of similar size, especially in contexts characterized by limited monitoring networks, providing a robust framework for the integrated assessment of pollution exposure.

### MODIS-MAIAC AOD data acquisition and processing

AOD data were obtained from the MODIS-MAIAC (MCD19A2) satellite products, associated with the Terra and Aqua satellites, distributed through the NASA Earth Observing System Data and Information System platform. The MCD19A2 product provides AOD values at a spatial resolution of 1 km, calculated using the MAIAC algorithm (27, 28), which uses lookup tables and explicitly accounts for surface bidirectional reflectance. In this study, AOD values at 0.550 nm (29), corresponding to daily MODIS observations, were used.

Data download was performed using the Earthdata Download application, developed by NASA Earth Science Data Systems. To ensure data quality, images were filtered by cloud cover, selecting only scenes with a maximum cloud cover of 30% in the study area. Pixels affected by cloud cover were removed during preprocessing and reclassified as NoData values in ArcGIS Pro 3.6 (Esri, Redlands, California, USA). Also, satellite images that could not be adequately corrected were removed from the analysis to avoid affecting the quality of observations.

Daily AOD values were aggregated at seasonal and annual levels by averaging over each pixel. Data were cropped at the OMA boundary and reprojected into a common coordinate system (Stereo_1970) for spatial analysis. AOD values were subsequently correlated with data from ground-based air quality monitoring stations to develop the PM₁₀ estimation model.

### Ground monitoring data and processing

To correlate PM₁₀ concentrations with AOD derived from satellite data, records from four air quality monitoring stations located within the OMA were used. These stations constitute the entire official air-quality monitoring network operating in Oradea and are managed by the National Agency for Environment and Protected Areas (Bihor County Environment Directorate), which provides standardized hourly measurements of PM₁₀ concentrations and MEs. The analysis focused on 2025, as this was the only year for which complete, continuous, and coherent datasets required for the simultaneous integration of PM₁₀, MEs, and satellite-derived AOD were available for all four monitoring stations. Despite the limited number of stations, their spatial distribution covers contrasting environments within the study area, including the central and densely urbanized part of Oradea, peripheral areas, highly trafficked and intensively used urban sectors, as well as a station located in a less urbanized environment with more rural characteristics (Figure 1). This spatial configuration provides observations representative of different urban and peri-urban settings and supports their use to develop the model and to extrapolate PM₁₀ estimates across the wider metropolitan area by integrating them with spatially continuous satellite-derived AOD and MEs.

The choice of PM₁₀ is justified by its relevance for local-scale analysis, as this fraction includes both fine particles (PM₂.₅) and coarse particles, making it more representative of urban pollution sources, such as road traffic, dust resuspension, or other local emissions. In addition, PM₁₀ also includes the PM₂.₅ component, recognized for its major impact on human health, thus providing a more comprehensive perspective on population exposure. At the same time, the use of PM₁₀ is also conceptually appropriate, given that AOD reflects the total aerosol load on the entire atmospheric column, being closer to the PM₁₀ fraction than to PM₂.₅, which represents only one component of it.

Given that the direct relationship between PM₁₀ and AOD is generally moderate, and PM₁₀ concentrations are the result of a combination of atmospheric and anthropogenic factors, it was decided to integrate AOD together with a set of MEs, namely air temperature (T), relative humidity (RH), solar radiation (SR), atmospheric pressure (AP) and wind speed (WS). These indicators were chosen for their direct influence on aerosol dynamics, particularly on the processes of dispersion, transport, and accumulation of PM in the lower atmosphere.

Although the empirical framework relies on a sparse network of four official air quality monitoring stations within the OMA, these stations are strategically distributed to capture distinct urban environmental typologies. Of the four stations analyzed, three are located in Oradea Municipality: station BH-1 is in a central area with intense road traffic; BH-2 is in the vicinity of the industrial area in the north-west of the city (CET I) and the garbage dump; and BH-3 is along a high-traffic artery. Station BH-4 is located outside the urban area, in a rural setting immediately adjacent to the municipality, thus providing a benchmark for conditions less influenced by urban activities. Thus, despite the limited number of monitoring stations, their spatial distribution captures a heterogeneous range of urban and peri-urban exposure conditions across the metropolitan area, enhancing the representativeness of the ground-based dataset.

Given that AOD is derived by combining MODIS Terra and Aqua observations taken during the day (approximately between 10:30 and 13:30 local time), ground-based data were aggregated between 10:00 and 14:00. Thus, for each day, the average of the values recorded during this interval was calculated in order to ensure the closest possible temporal correspondence between satellite and *in situ* observations.

### Socio-spatial data acquisition and processing

To identify areas within the OMA prone to pollution, a composite index was developed by integrating a series of SSIs relevant to population exposure. These include estimated PM_10_ values based on AOD (E_PM₁₀_), population density (Pop) and built-up area density (BuildD), distance from hotspots characterized by high E_PM₁₀_ values (DistHS), as well as the characteristics of the built environment, expressed by the height and function of buildings (BuildH&F). Environmental indicators, such as the Normalized Difference Vegetation Index (NDVI) and land elevation (LE), were also included. All of these parameters play a decisive role in shaping an exposure index, reflecting both the potential level of pollution and the environment’s capacity to favor or reduce the dispersion of pollutants and, implicitly, the population’s exposure.

Data for the Pop, BuildD, and BuildH&F indicators were obtained as 100 × 100 m-resolution rasters from the Joint Research Centre of the European Commission (30). Subsequently, the datasets were aggregated at the OMA level and corrected by integrating field observations and official census data to ensure a more faithful representation of the spatial distribution of the population and the characteristics of the built environment. E_PM₁₀_ represents the seasonal mean spatial distribution of PM₁₀ concentrations, obtained by correlating AOD values with MEs and PM₁₀ measurements made at the monitoring stations, based on statistical models developed in the study. As for LE, it was derived from a digital elevation model obtained from the ALOS World 3D (AW3D) dataset, provided by JAXA.

NDVI was derived from Sentinel-2 satellite images provided by the Copernicus program, using the red (B4) and near-infrared (B8) spectral bands, at a spatial resolution of 10 m. For the analysis, seasonal and annual average NDVI values were calculated to capture the temporal variability of vegetation. NDVI was calculated according to the relationship:


$$\mathrm{NDV}I_{\text{seasonal}/\text{annual}}=\frac{1}{n}\sum_{i=1}^{n}(\frac{\mathrm{NI}R_{i}-\mathrm{RE}D_{i}}{\mathrm{NI}R_{i}+\mathrm{RE}D_{i}})$$

where *NIR* is the near-infrared reflectance, *RED* is the red band reflectance, *i* represents each observation within the season or year, and *n* is the total number of observations in the selected period.

### Statistical model development for estimating PM₁₀ concentrations

To analyze the relationship between AOD and PM₁₀ concentrations, a multiple linear regression model was applied. The choice of this type of model is based on its ability to simultaneously account for the effects of multiple explanatory variables, given that PM₁₀ concentrations result from a combination of atmospheric and anthropogenic factors and that the direct relationship with AOD is generally moderate.

The distribution of PM₁₀ values presented a pronounced asymmetry, which is why a logarithmic transformation was applied in order to stabilize the variance and improve the performance of the model:


$$\log(PM_{10}+1)$$

The general form of the regression model used is:


$$\log(PM_{10}+1)=\beta_{0}+\sum_{i=1}^{5}\beta_{1}X_{i}$$

where PM_10_ represents the concentration of PM measured on the ground with a diameter of 10 μm or smaller expressed in μg/m^3^, *X_i_* represent the explanatory variables of the model, considered predictors of *PM₁₀* concentrations, each corresponding to a MEs relevant for the processes of PM formation, dispersion and accumulation (AOD, T, RH, SR, AP, WS), *β0* is the intercept of the model and represents the estimated value of log(PM₁₀ + 1) when all explanatory variables are equal to zero, $\beta_{i}$ is the regression coefficient that quantifies the effect of each variable *X_i_* on the logarithm of PM₁₀.

Given that MEs were only available at monitoring stations and not as continuous spatial fields, generalization across the study area was necessary. Thus, for each season, the average values of the meteorological elements were determined and subsequently treated as constant variables across the entire study area.

By substituting the seasonal average values of MEs in the initial model, a simplified form of the relationship was obtained, of the type:


$$\log(PM_{10}+1)=(\beta_{0}+\sum_{i=2}^{5}\beta_{i}\bar{X_{i}}\text{season})+\beta_{1}\times \mathrm{AOD}.$$

where *PM_10_* is the concentration of PM measured at ground level with a diameter of 10 μm or smaller, expressed in μg/m^3^, $\beta_{0}$ is the free term of the model (the intercept), which defines the baseline level of the dependent variable, $\beta_{1}$ the regression coefficient associated with the AOD variable, *β_i_* regression coefficients corresponding to MEs included in the model, $X_{i}$ represents explanatory MEs, $\bar{X_{i}}\text{season}$ is the seasonal average value of each ME, calculated for the entire study area, and AOD is the optical depth of aerosols throughout the atmospheric column, derived from MODIS-MAIAC satellite data.

This reformulation implies a conceptual separation between the spatial and atmospheric components of the model. Thus, the spatial variability of PM₁₀ concentrations is mainly attributed to the AOD distribution, which serves as a proxy for atmospheric aerosol loading, while the influence of MEs is represented by an aggregate term that remains constant across the entire area for each season. Through this approach, a spatial generalization of the model is achieved, given that MEs are not available as continuous fields. Consequently, the model enables estimation of the spatial distribution of PM₁₀ solely from AOD variability, while preserving the average influence of MEs on background concentration levels. This formulation was applied to each raster cell corresponding to daily AOD values, separately for each season, to estimate the spatial distribution of PM₁₀ concentrations. Thus, for each day, the model was implemented pixel by pixel, using the available AOD values, while the term $C_{\text{season}}$ was held constant for the corresponding season.

To return the estimated values to the physical domain of concentrations (μg/m^3^), the inverse transformation of the logarithmic function used in the modelling stage was applied. Thus, the return from the logarithmic space to the original space of the dependent variable was achieved, according to the relationship:


$$PM_{10}=\exp(\beta_{0}+\sum_{i=2}^{5}\beta_{i}\bar{X_{i}}\text{season}+\beta_{1}\times \mathrm{AOD})-1$$

This transformation reflects the nonlinear relationship between the variables, enabling the conversion of estimates from the log-linear model into physically interpretable values. The term “−1” compensates for the initial transformation applied to the dependent variable, ensuring the mathematical consistency of the model and avoiding overestimation of values at low concentration levels.

The evaluation of model performance against *in situ* observations revealed a systematic tendency to underestimate PM₁₀ concentrations, more pronounced in the cold season. This deviation can be attributed both to the inherent limitations of AOD as a proxy for PM_10_ concentrations and to the variability in local emission and dispersion processes, which the initial model insufficiently captures.

To correct this systematic deviation, a seasonal correction term was determined $B_{\text{season}}$ , defined as an estimator of the average bias between observed and modelled values. For each season, the bias was calculated as the mean difference between observed and predicted PM₁₀ values. The resulting seasonal correction term was then added to the corresponding predicted values. The corrected estimates were subsequently evaluated using RMSE, MAE, MAPE, MBE, and Leave-One-Out Cross-Validation (LOOCV) to assess the model’s predictive performance and the risk of overfitting. This term reflects the systematic deviation of the model depending on the specific conditions of each season and allows the adjustment of the background level of the estimates by:


$$PM_{10}=\exp(\beta_{0}+\sum_{i=2}^{5}\beta_{i}\bar{X_{i}}\text{season}+\beta_{1}\times \mathrm{AOD})-1+B_{\text{season}}$$

where *B_season_* represents the seasonal correction term, defined as the average estimated bias for each season, calculated based on the differences between observed and modelled values of PM₁₀ concentrations.

The model was implemented in an explicit spatial framework at the pixel level using raster processing tools in ArcGIS Pro 3.6 (Esri, Redlands, California, USA). The approach involved applying the model function to each grid cell, thereby enabling continuous estimation of the spatial distribution of PM₁₀ concentrations.

For each season, AOD values were used as the input spatial variable, while constant seasonal terms were integrated uniformly over the entire study area. Thus, for each daily raster, the model was applied pixel-wise to generate continuous spatial fields of E_PM₁₀_ concentrations. This approach allows the transposition of the AOD–PM₁₀ statistical relationship into a spatial context, facilitating the analysis of intra-urban and peri-urban variability in air pollution and the generation of detailed maps of concentration distribution across the entire OMA.

To evaluate the predictive performance of the proposed multiple linear regression model, several statistical validation metrics were computed, including the Root Mean Square Error (RMSE), Mean Absolute Error (MAE), Mean Absolute Percentage Error (MAPE), and Mean Bias Error (MBE). These indicators were used to quantify the average prediction error, the relative prediction accuracy, and the presence of any systematic overestimation or underestimation of PM₁₀ concentrations, according to the following equations:


$$\text{RMSE}=\sqrt{\frac{1}{n}}\sum_{i=1}^{n}{\left(O_{i}-P_{i}\right)}^{2}$$

$$\mathrm{MAE}=\frac{1}{n}\sum_{i=1}^{n}|O_{i}-P_{i}|$$

$$\text{MAPE}=\frac{100}{n}\sum_{i=1}^{n}|\frac{O_{i}-P_{i}}{O_{i}}|$$

$$\mathrm{MBE}=\frac{1}{n}\sum_{i=1}^{n}(O_{i}-P_{i})$$

where *O_i_* is the observed PM₁₀ concentration, *P_i_* is the predicted PM₁₀ concentration, and *n* is the total number of observations.

In addition, a LOOCV procedure was performed to assess the robustness and generalization capability of the model. It was performed by iteratively excluding one observation from the dataset, fitting the regression model using the remaining observations, and predicting the excluded observation, as follows:


$${\hat{y}}_{i}^{\left(-i\right)}=f_{-i}(X_{i})$$

where ${\hat{y}}_{i}^{\left(-i\right)}$ is the predicted value for observation *i*, $\;f_{-i}$ represents the regression model trained on all observations except observation *i,* and $X_{i}$ is the vector of predictors for observation *i*.

This procedure was repeated for all observations, ensuring that each sample was used once for validation. The prediction errors from all iterations were subsequently used to compute the RMSE, MAE, MAPE, and MBE for the cross-validation procedure. This process was repeated for all observations, providing an independent evaluation of the model performance and reducing the risk of overfitting.

### Construction of the composite PM_10_ spatial exposure index

The assessment of the potential impact of population exposure to PM_10_ does not depend exclusively on the spatial distribution of PM_10_ concentrations but is determined to a significant extent by the characteristics of the built environment and, in particular, by the distribution of the exposed population. Thus, high PM_10_ levels in densely populated areas imply a significantly higher risk to human health than similar values in sparsely populated areas, such as agricultural or rural areas with few or no inhabitants.

Therefore, the exposure analysis cannot be conducted solely using absolute PM_10_ concentrations. It must also integrate factors that reflect the population’s spatial susceptibility to this type of contamination. In this context, a composite index was developed by integrating several relevant SSI-type indicators to capture spatial variations in the population’s exposure to PM_10_ at the OMA level.

The proposed index enables the spatial identification of areas characterized by the convergence of high values of E_PM₁₀_ and the variables Pop, BuildD, DistHS, BuildH&F, NDVI, and LE, thereby creating favorable conditions for the accumulation, recirculation, and stagnation of PM_10_. This complex interaction of factors consequently increases the potential for exposure and amplifies the negative impact on human health.

Each spatial variable analyzed was divided into its own number of classes $k_{i}$ , depending on the distribution of the data and their significance, as follows:


$$X_{i}\to \{C_{i1},C_{i2},\ldots ,C_{ik_{i}}\}$$

where $X_{i}$ is the initial variable, $C_{\mathrm{ij}}$ is the class *j* of variable *i*, $k_{i}$ is the number of classes (variable from one indicator to another).

This discretization stage was necessary to transform heterogeneous variables, expressed in different units and scales, into a standardized form, which would allow their comparison and integration into a common framework of analysis and confer different scores to the classes, depending on the importance of each interval of each $X_{i}$ within the created model.

Thus, to each class $X_{i}$ created was assigned a normalised score $s_{\mathrm{ij}}$ , expressed in the interval [0,1], reflecting its overall contribution to the spatial exposure of PM_10_ contamination on population health. The closer the scores assigned to the classes are to the maximum value of the interval, the higher their contribution to the model, reflecting a potentially greater impact of PM_10_ exposure. The class scoring process can be expressed mathematically as follows:


$$S_{i}(p)=s_{\mathrm{ij}},\text{if}\;X_{i}(p)\in C_{\mathrm{ij}}$$

where $S_{i}(p)$ is the score of pixel *p* in layer *i*, $s_{\mathrm{ij}}$ is the score associated with class *j*, p is the spatial position of the pixel containing the values.

In addition to the class score ( $s_{\mathrm{ij}}$ ), each variable contributes to the final index and through the overall weight of the layer ( $w_{i}$ ), which represents the effective contribution of each variable $X_{i}$ analysed at the pixel level, within a normalised interval [0,1], by:


$$w_{i}\cdot S_{i}(p)$$

where $w_{i}$ is the relative importance of the layer in the model and $S_{i}(p)$ is the reclassified score of the pixel. Variables $X_{i}$ considered to have a greater influence on exposure were given higher weights in the model, while indicators with a relatively low impact on the final exposure were assigned lower weights.

Finally, the composite index was calculated by aggregating all weighted variables and can be expressed mathematically as follows:


$$CI_{PM_{10}}(p)=\sum_{i=1}^{7}w_{i}\cdot S_{i}(p)$$

and in the explicit form used within the model:


$$\begin{array}{c} CI_{PM_{10}}(p)=0.40\cdot S_{\mathrm{EP}M_{10}}(p)+0.20\cdot S_{\text{DistHS}}(p)+0.15\cdot S_{\mathrm{Pop}}(p)\\ +0.10\cdot S_{\text{BuiltD}}(p)+0.05\cdot S_{\text{NDVI}}(p)\\ +0.05\cdot S_{\mathrm{LE}}(p)+0.05\cdot S_{\text{BuildH}\&F}(p) \end{array}$$

where S_EPM₁₀_*(p). SB*_uildH&F_*(p)* represent the values per class for each analysed indicator, and 0.40.0.0.05 represent the weight indices within the normalized interval [0,1].

In its original form, the model was used to estimate annual values of probable exposure to PM_10_. To capture seasonal variability, the formula was recalibrated by introducing seasonally dependent components, as follows:


$$\begin{array}{c} CI_{PM_{10}}^{\left(s\right)}(p)=0.40\cdot S_{\mathrm{EP}M_{10}}^{\left(s\right)}(p)+0.20\cdot S_{\text{DistHS}}^{\left(s\right)}(p)+0.15\cdot S_{\mathrm{Pop}}(p)\\ +0.10\cdot S_{\text{BuiltD}}(p)+0.05\cdot S_{\text{NDVI}}^{\left(s\right)}(p)\\ +0.05\cdot S_{\mathrm{LE}}(p)+0.05\cdot S_{\text{BuildH}\&F}(p) \end{array}$$

where $S_{\mathrm{EP}M_{10}}^{\left(s\right)}$ , $S_{\text{DistHS}}^{\left(s\right)}$ and $S_{\text{NDVI}}^{\left(s\right)}$ represent the simplified values, normalised to the [0,1] interval, of the values recorded for each season.

The weighting scheme for the composite exposure index was established based on the relative importance of each variable in determining human exposure to PM₁₀, informed by previously published air pollution exposure assessment frameworks and complemented by sensitivity tests performed by the authors specifically for the present study. The highest weight was assigned to E_PM₁₀_, as it represents the primary pollutant directly associated with human exposure. Intermediate weights were assigned to POP and distance-related variables, reflecting both the spatial distribution of the population and the influence of DistHS and BuildD, while lower weights were assigned to building features (BuildH&F) and environmental variables (NDVI and LE), which mainly affect pollutant dispersion, deposition, and microclimatic conditions rather than directly determining exposure levels. This weighting strategy is consistent with previous exposure assessment studies that integrate pollutant concentrations with demographic and environmental variables to characterize population exposure (31–33).

To evaluate the robustness of the proposed weighting scheme, a one-at-a-time sensitivity analysis was performed by independently varying the weight assigned to each indicator by ±20%, while proportionally adjusting the remaining weights to maintain a total weight of 1. The recalculated composite exposure indices remained highly correlated with the original index in all scenarios. The largest sensitivity was observed for EPM₁₀, the dominant component of the index, with correlation coefficients ranging from 0.872 to 0.948, whereas DistHS showed the second-largest influence (r = 0.971–0.980). The comparatively greater sensitivity to EPM₁₀ and DistHS is consistent with their higher assigned weights and their variability within the study area, which result in a stronger contribution to changes in the composite index when their weights are modified. Variations in the remaining indicators produced only negligible changes, with correlation coefficients generally exceeding 0.99. These results indicate that the proposed weighting scheme is robust and the composite index remained highly consistent with the original configuration, preserving the overall spatial exposure pattern. The consistently high correlation coefficients indicate that the index is not overly sensitive to the choice of individual weights.

Regarding the final values of the composite index $IC_{\mathrm{PM}10}$ , it is important to note that:


$$0\leq S_{i}(p)\leq 1\;\text{and}\sum w_{i}=1$$

which results in:


$$0\leq IC_{PM_{10}}(p)\leq 1$$

where values approaching 0 indicate areas with low predisposition, while values approaching 1 indicate areas with high predisposition to PM₁₀ contamination, implying a greater potential impact on human health.

By including the E_PM₁₀_ variable, the index acquires a hybrid character, integrating both explanatory variables and a dependent component represented by the modelled spatial distribution of the pollutant. In this context, the index can be considered a composite indicator of spatial predisposition, combining the causal and descriptive dimensions of PM_10_ contamination, going beyond the conceptual framework of theoretical susceptibility.

## Results

### Spatiotemporal variability of MODIS-MAIAC aerosol optical depth

Statistical analysis of AOD values highlights seasonal variations at the OMA level. The seasonal mean values indicate a maximum in the summer season (0.189 ± 0.017), followed by winter (0.145 ± 0.014), spring (0.135 ± 0.009) and autumn (0.092 ± 0.010). The annual mean value of AOD is approximately 0.140 ± 0.008.

The distribution of values shows moderate variability, with well-defined intervals for each season. In the summer season, AOD reaches its highest values, ranging from 0.143 to 0.237. In contrast, the autumn season presents the lowest values (0.068–0.121), indicating atmospheric conditions more favorable to dispersion or a reduction in emission sources. The transitional seasons (spring and autumn) show lower variability than summer and winter, as reflected in the lower standard deviations. During winter, although the mean values are lower than in summer, the range of variation (0.116–0.183) and the relatively high dispersion suggest the possible influence of local factors, such as residential heating and stable atmospheric conditions.

**Figure 3:**
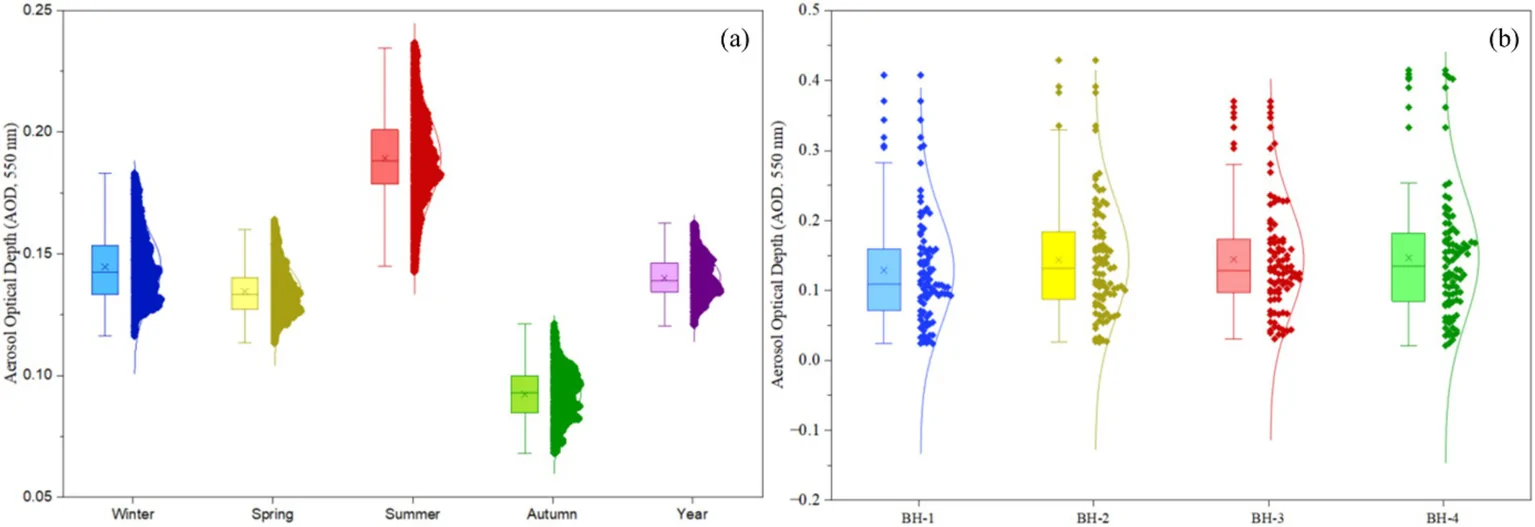
Distribution of MODIS-MAIAC Aerosol Optical Depth (AOD) values for 2025: **(a)** Seasonal and annual patterns at the level of Oradea Metropolitan Area, and **(b)** Variability across air quality monitoring stations within the Oradea Municipality.

The analysis of the percentile distribution indicates that approximately 50% of the data (median) is concentrated around 0.188 in summer, 0.143 in winter, 0.133 in spring, and 0.093 in autumn. Also, the relatively narrow interquartile ranges indicate a compact distribution of AOD values, with infrequent extreme variations (Figure 3a).

Analysis of the AOD values extracted at the four monitoring stations reveals moderate spatial variability. The mean values are relatively close, ranging between 0.129 (BH-1) and 0.147 (BH-4), with slightly higher values for BH-2 and BH-3, and the standard deviations (≈0.08–0.09) indicate significant temporal dynamics of aerosols.

The maximum values highlight episodes of high atmospheric loading, especially for BH-2 (0.429) and BH-4 (0.415), while the minimum values are low for all stations (≈0.02–0.03). The median values fall between 0.108 and 0.134, suggesting a relatively stable distribution, without frequent extremes (Figure 3b). Urban stations (BH-2 and BH-3), associated with heavy traffic and industrial activities, show slightly higher values, while the rural station BH-4 records the highest average value. Overall, spatial differences are moderate, indicating that local sources and regional processes jointly control AOD distribution.

**Figure 4:**
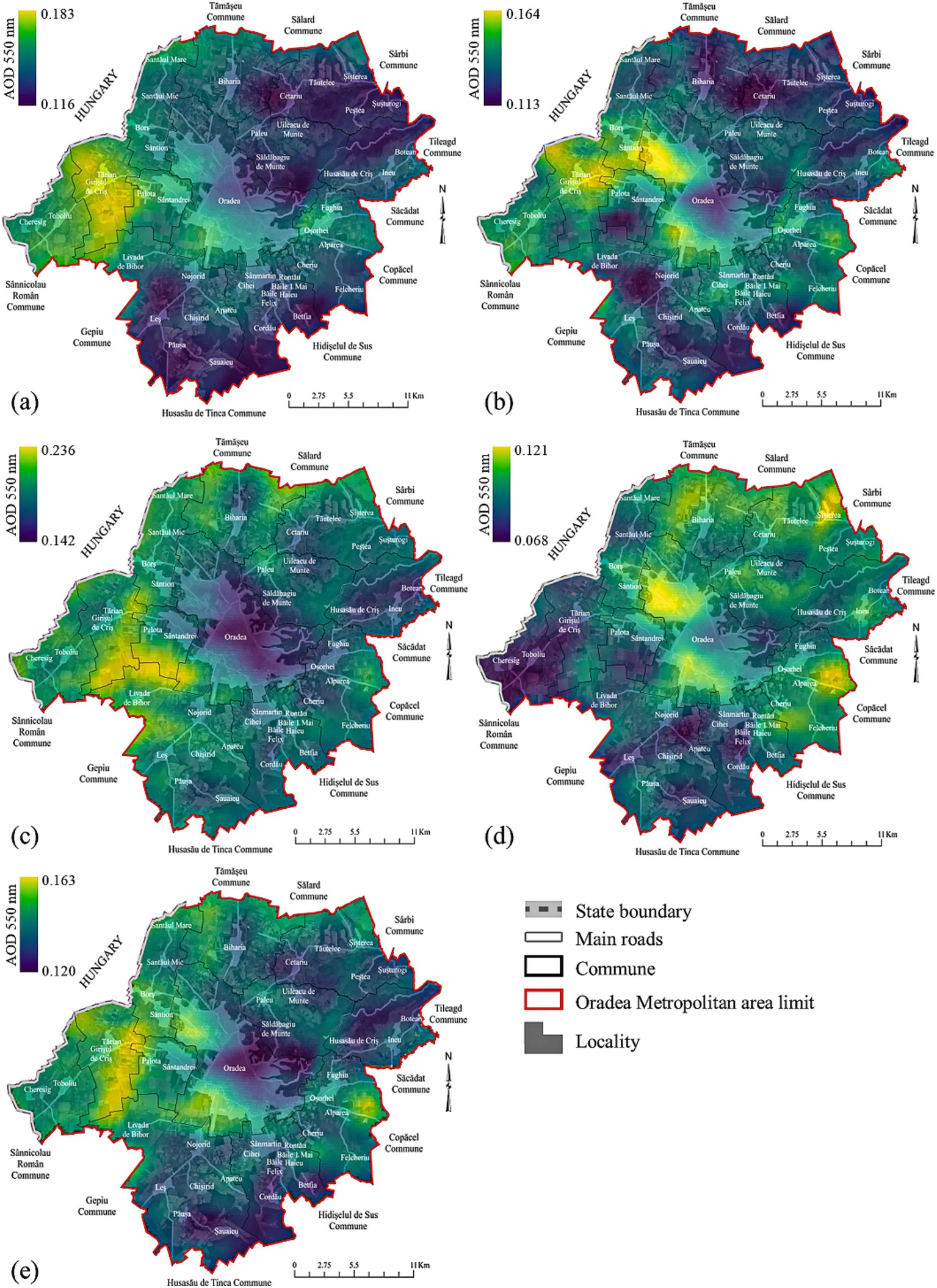
Spatial distribution of mean aerosol optical depth (AOD) derived from MODIS-MAIAC for 2025, illustrating seasonal averages and the annual mean across the study area: **(a)** Winter; **(b)** Spring; **(c)** Summer; **(d)** Autumn; **(e)** Annual mean.

The analysis of the spatial distribution of AOD highlights a coherent seasonal pattern, characterized by the concentration of high values in the western sector of the analysed area. In the winter season, the maximum values, up to 0.183, are predominantly located in this area, a similar behavior being observed in the spring (maximum 0.164) and in the summer season (maximum 0.237). These outbreaks of maximum intensity are consistently associated with the administrative-territorial units of Sântandrei and Girisu de Criș, suggesting a possible influence of local conditions favorable to aerosol accumulation or of regional transport. In contrast, in the autumn season, the distribution of high AOD values becomes more spatially dispersed, with approximately six distinct nuclei exceeding the 0.1 threshold, unevenly distributed across the entire OMA. At the same time, high values of AOD are constantly recorded, especially in the winter, spring and autumn seasons, above the main industrial areas in Oradea Municipality, namely industrial zone II (southern area) and CET I (northwestern area), where values frequently exceed 0.12. These concentrations may highlight a contribution from industrial sources to atmospheric aerosol loading (Figure 4).

Low AOD values are predominantly associated with hilly areas and the southern sector of the OMA, characterized by rural areas or natural vegetation cover of the land. Also, the central area of Oradea Municipality has relatively low values in all seasons, although it is characterized by a very high degree of urbanization, with impervious surfaces and heavy traffic.

### Aerosol optical depth-based estimation of PM₁₀ concentrations using meteorological elements

**Figure 5:**
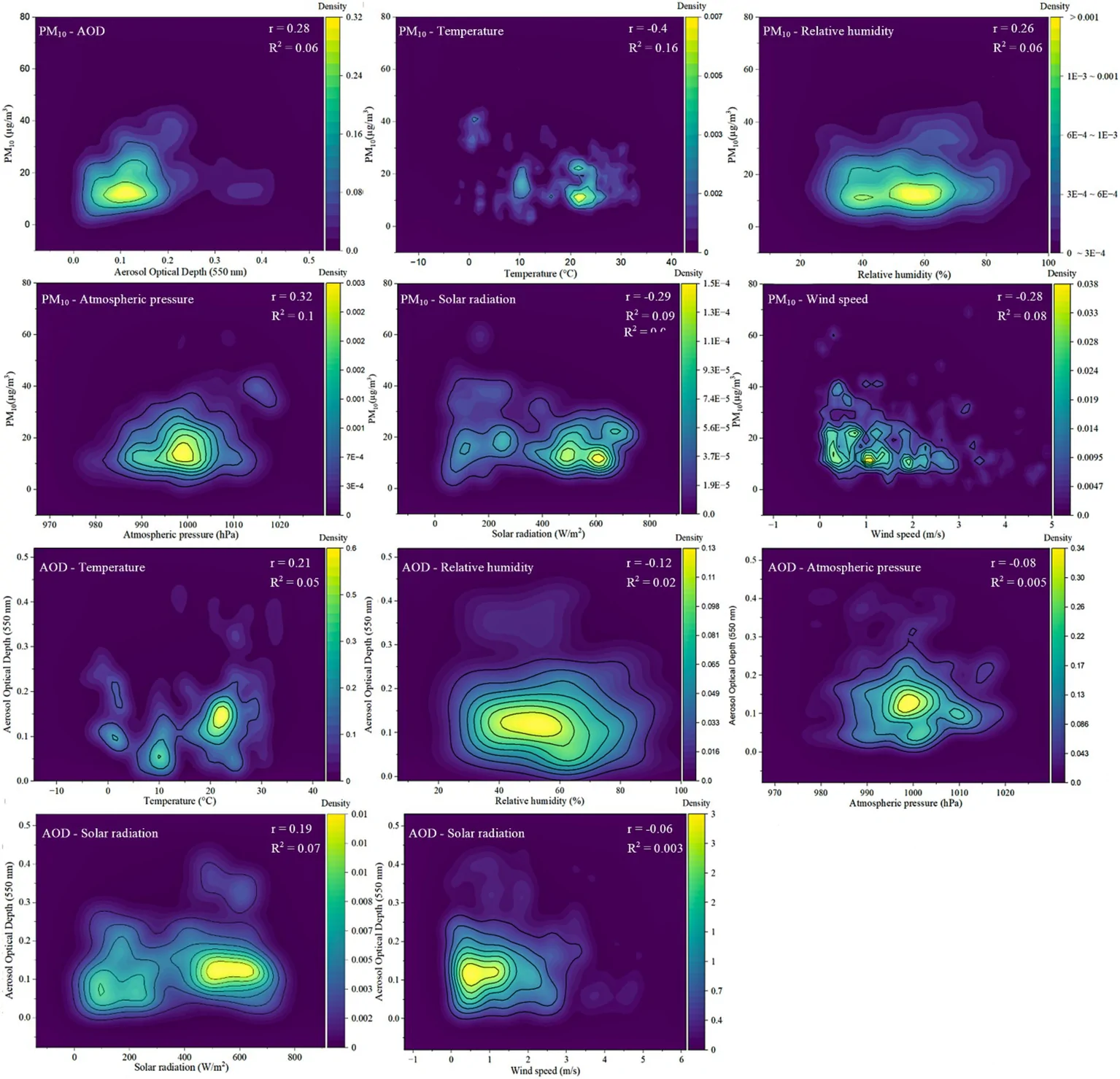
Kernel density-based analysis of the relationships between PM_10_, AOD and MEs, highlighting the variability and strength of correlations across different atmospheric conditions.

The analysis of the relationships among PM₁₀ concentrations, AOD, and MEs reveals moderate yet physically consistent dependencies. The correlation between PM₁₀ and AOD is positive but relatively weak (*r* = 0.28, *R*^2^ = 0.06), confirming that AOD is a useful but incomplete proxy for PM₁₀. Among MEs, T shows the strongest relationship with PM₁₀ (*r* = −0.40, *R*^2^ = 0.16), indicating a tendency for PM_10_ concentrations to decrease with increasing T, probably as a result of enhanced vertical dispersion and mixing processes. WS (*r* = −0.28, *R*^2^ = 0.08) and SR (*r* = −0.29, *R*^2^ = 0.09) also show negative correlations, suggesting a role in reducing PM_10_ accumulation through ventilation and atmospheric instability (Figure 5).

In contrast, AP shows a moderate positive correlation (*r* = 0.32, *R*^2^ = 0.10), indicating an association between high PM₁₀ values and stable atmospheric conditions (especially anticyclonic conditions) that favor air mass stagnation. RH has a weak positive correlation (*r* = 0.26, *R*^2^ = 0.06), suggesting a possible contribution of hygroscopic processes and secondary aerosol formation. The relationships between AOD and MEs are generally weaker, with low values of the coefficient of determination, indicating that AOD variability is influenced not only by local conditions but also by regional processes and atmospheric transport. However, a weak positive correlation is observed between AOD and T (*r* = 0.21) and SR (*r* = 0.19), and very weak negative correlations with RH (*r* = −0.12) and WS (*r* = −0.06) (Figure 5). The density distributions highlight the concentration of PM₁₀ values in the range of 5–20 μg/m^3^, which is mainly associated with AOD values between 0.05 and 0.15. At the same time, a greater dispersion of PM₁₀ values is observed at moderate AOD values, confirming the determining influence of MEs on *in situ* concentrations. Overall, the results justify the use of a multiple regression model for PM_10_ estimation, in which AOD is integrated together with MEs. The weak direct AOD–PM_10_ correlation highlights the need to account for atmospheric factors to capture the dispersion, transport, and accumulation processes of PM10, an essential aspect for obtaining realistic spatial estimates.

The PM_10_ performance evaluation indicates an overall satisfactory predictive capability of the proposed regression model. For the complete dataset, the model achieved an *R*^2^ of 0.35, RMSE of 7.8 μg/m^3^, an MAE of 2.9 μg/m^3^, a MAPE of 44.6%, and an MBE of −0.44 μg/m^3^, indicating only a slight tendency to overestimate PM₁₀ concentrations (Table 1). To evaluate the overall predictive performance and stability of the proposed regression model, and to assess potential overfitting due to sample size, the baseline model performance was compared with LOOCV results. Under LOOCV, the corresponding values were *R*^2^ = 0.28, RMSE = 8.16 μg/m^3^, MAE = 4.36 μg/m^3^, MAPE = 48.01%, and MBE = 0.10 μg/m^3^ (Table 2). The relatively limited changes in error magnitude between the baseline and cross-validated results indicate that the model maintains a reasonably stable predictive performance when applied to unseen observations, providing additional evidence against substantial overfitting.

**Table 1:** Performance evaluation of the proposed PM_10_ estimation model based on statistical validation metrics (RMSE, MAE, MAPE, and MBE) for each monitoring station, seasonal subsets, the complete dataset, and LOOCV.

Air Quality Station /Season	RMSE (μg/m^3^)	MAE (μg/m^3^)	MAPE (%)	MBE (μg/m^3^)
BH-1	9.2	5.4	52	−0.15
BH-2	8.1	3.6	36.2	−1.01
BH-3	8.1	2.1	37.1	−0.44
BH-4	5.8	0.59	53.4	−0.16
Winter	8.7	3.6	86.7	−0.36
Spring	7.8	3.1	34.7	−0.44
Summer	7.4	2.5	32.4	−0.45
Autumn	7.3	2.4	24.7	−0.5

**Table 2:** Comparative performance metrics of the baseline multiple linear regression model and the Leave-One-Out Cross-Validation (LOOCV) framework for PM_10_ estimation.

Model evaluation framework	*R*^2^	RMSE (μg/m^3^)	MAE (μg/m^3^)	MAPE (%)	MBE (μg/m^3^)
Baseline multiple linear regression	0.35	7.8	2.9	44.6	−0.44
LOOCV	0.28	8.16	4.36	48.01	0.1

The seasonal analysis revealed that the largest prediction errors occurred in winter (MAPE = 86.7%), whereas the best performance was observed in autumn (MAPE = 24.7%), followed by summer (MAPE = 32.4%) and spring (MAPE = 34.7%). The reduced accuracy during winter is consistent with the greater complexity of atmospheric processes, including increased residential heating emissions, more frequent thermal inversions, lower planetary boundary layer heights, and greater temporal variability in aerosol vertical distribution. These conditions weaken the relationship between column-integrated AOD and ground-level PM₁₀ concentrations, leading to greater prediction uncertainty regardless of the modelling approach employed. In contrast, the more homogeneous atmospheric conditions during the warm seasons contribute to a stronger correspondence between satellite-derived AOD and surface PM₁₀ concentrations. The station-based validation showed some spatial variability in model performance. BH-4 presented the lowest RMSE (5.8 μg/m^3^) and MAE (0.59 μg/m^3^), while BH-1 exhibited the largest prediction errors (RMSE = 9.2 μg/m^3^; MAPE = 52%). These differences likely reflect local emission sources, heterogeneous urban morphology, and the influence of micro-scale meteorological conditions, which cannot be fully captured by the spatial resolution of Sentinel-5P observations and the simplified linear regression model. Nevertheless, the consistently low MBE values across all monitoring stations (ranging from −1.01 to −0.15 μg/m^3^) indicate the absence of a pronounced systematic bias (Table 1).

**Figure 6:**
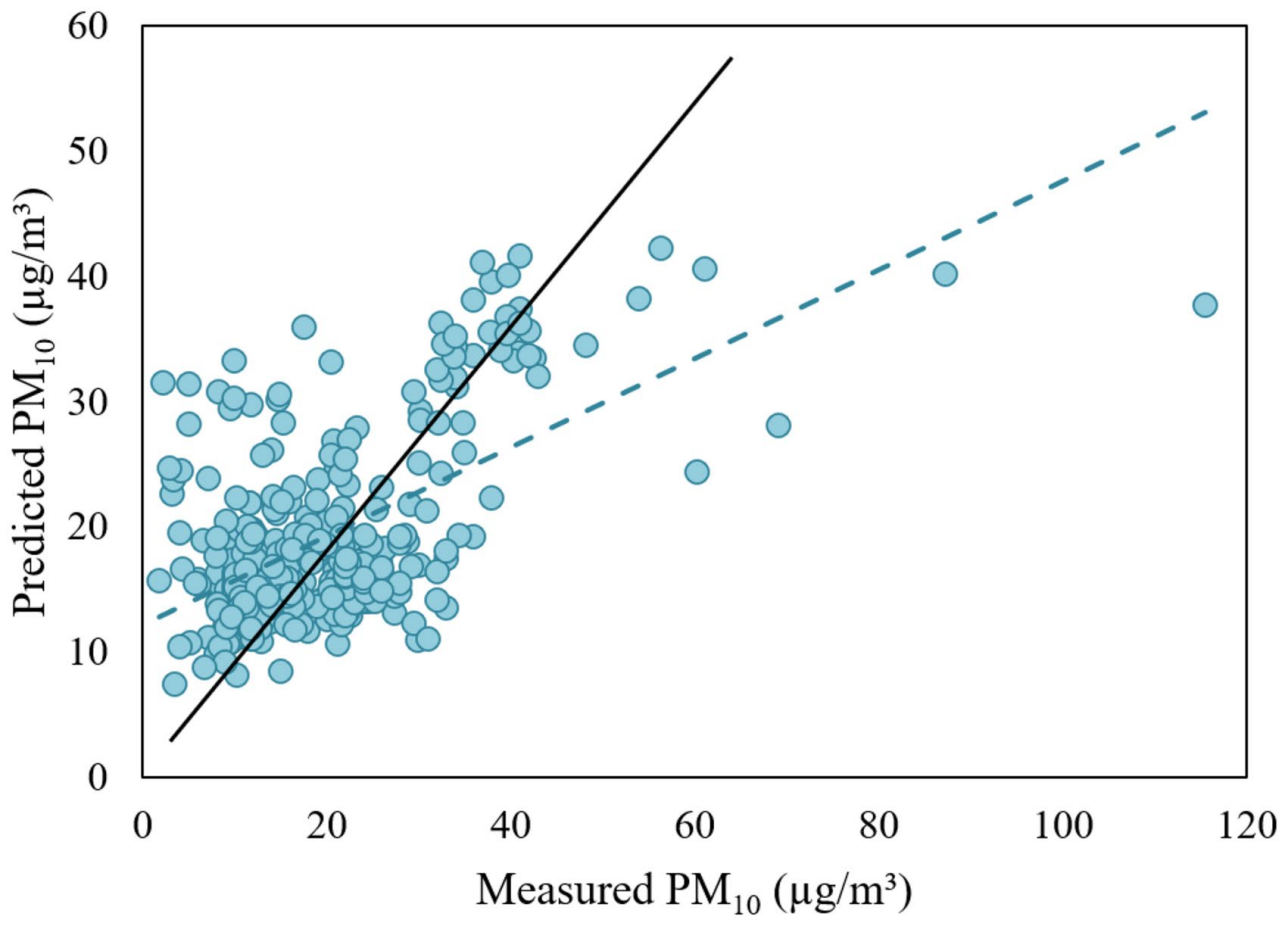
Scatter plot illustrating the relationship between measured PM_10_ at all air quality monitoring stations and e_pm10_ concentrations.

The scatter plot comparing the observed PM₁₀ and E_PM₁₀_ concentrations indicates a moderate agreement between the measured and estimated values. Most observations are distributed around the 1:1 agreement line, confirming that the proposed regression model reproduces the general variability of PM₁₀ without exhibiting a pronounced systematic bias (Figure 6). Nevertheless, greater point dispersion is observed at higher PM₁₀ concentrations, indicating increased prediction uncertainty during pollution episodes with elevated PM levels. This behavior is expected, given the moderate relationship between satellite-derived AOD and ground-level PM₁₀, the influence of MEs, and the limited spatial coverage of the ground monitoring network.

Statistical analysis of E_PM₁₀_ values highlights clear seasonal variations at the OMA level, reflecting the influence of MEs and season-specific emission sources. The seasonal mean values indicate a pronounced maximum in the winter season (31.73 ± 0.56 μg/m^3^), followed by summer (17.17 ± 0.52 μg/m^3^), autumn (16.56 ± 0.31 μg/m^3^) and spring (16.45 ± 0.29 μg/m^3^). The annual mean value is approximately 20.48 ± 0.28 μg/m^3^, which indicates a dominant influence of the high values in the cold season on the general mean.

**Figure 7:**
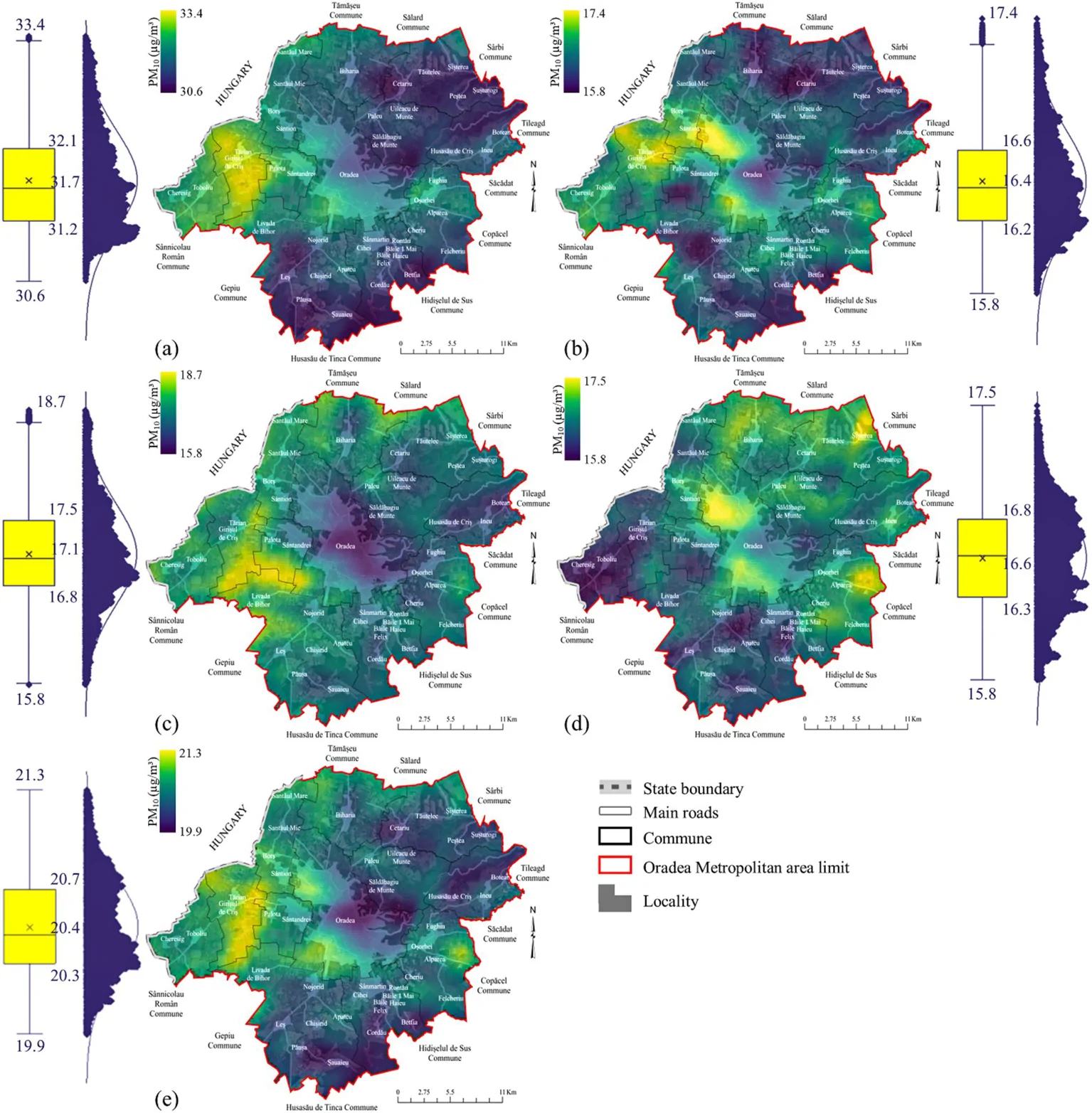
Spatial distribution of E_PM₁₀_ mean derived based on AOD MODIS-MAIAC and meteorological elements values for 2025, illustrating seasonal averages and the annual mean across the study area: **(a)** Winter; **(b)** Spring; **(c)** Summer; **(d)** Autumn; **(e)** Annual mean.

The distribution of the mean values shows a pronounced variability between seasons. During winter, the range of variation is the highest (approximately 30.6–33.4 μg/m^3^), a pattern also supported by the absolute extreme values, which reach a maximum of 41.8 μg/m^3^ and a minimum of 16.2 μg/m^3^. During the summer season, PM_10_ values vary between approximately 15.8 and 18.7 μg/m^3^. Although the mean is lower than in winter, the relatively high standard deviation suggests a more pronounced seasonal variability than in the transitional seasons, as confirmed by the wider absolute range (13.6–32.1 μg/m^3^). Spring and autumn show similar values (16.5 ± 0.29 μg/m^3^ and 16.6 ± 0.31 μg/m^3^), with relatively narrow ranges (15.8–17.5 μg/m^3^). However, the extreme values highlight the existence of episodes with high punctual values: in spring between 8.9 and 31.8 μg/m^3^, and in autumn between 9.6 and 27.3 μg/m^3^. The reduced variability is also confirmed by the lower values of the standard deviation (Figure 7).

The spatial distribution of E_PM₁₀_ within the analyzed area highlights a coherent pattern, influenced by both anthropogenic sources and seasonal meteorological conditions. In the cold season, the highest values are concentrated in the western sector of the OMA, suggesting a sharp accumulation of pollutants under stable atmospheric conditions. In the transitional seasons and in the warm one, the distribution becomes possibly more closely linked to industrial activities. In spring, higher values are observed in the main industrial areas of Oradea and in the western part of the OMA. In summer, they are maintained in the west and the southern industrial area; in autumn, they extend to both industrial areas, as well as to the north and east of the OMA. At the annual level, a predominance of high values is observed in the western area, particularly around the two large industrial sites (industrial area II and CET I) in Oradea Municipality, indicating a possible cumulative effect of anthropogenic emissions and regional pollutant transport. Beyond industrial emissions, a potentially significant source of PM₁₀ is the sustained construction activity across the urban area. These sites, often located near the two industrial zones identified in 2025, may contribute to elevated concentrations through dust resuspension and direct PM emissions, with possible cumulative effects (Figure 7).

### Identification of population exposure hotspots and air quality risk areas

**Figure 8:**
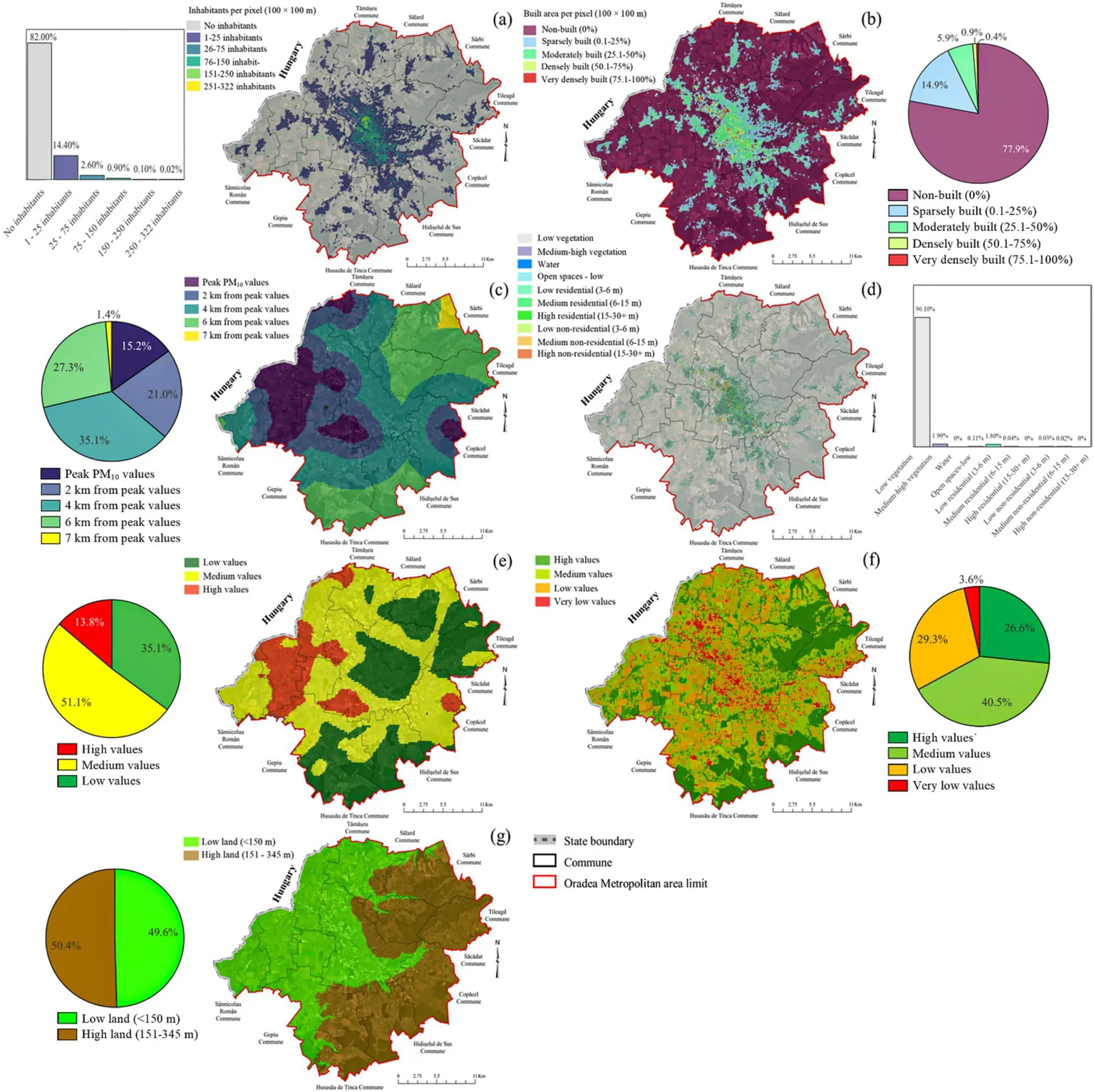
Spatial distribution of the annual mean values of the socio-spatial indicators underpinning the weighted multi-criteria composite index used to estimate the potential health impact of PM_10_ across the Oradea Metropolitan Area in 2025 [**(a)** Pop; **(b)** BuildD; **(c)** DistHS; **(d)** BuildH&F; **(e)** E_PM10_; **(f)** NDVI; **(g)** LE].

The spatial distribution of the indicators used to construct the composite index of PM₁₀ exposure highlights pronounced heterogeneity in MEs and SSIs at the OMA level (Figure 8). SSIs reflecting direct exposure, namely E_PM₁₀_, Pop, and BuildD, show clear spatial convergence in the Oradea Municipality and in peri-urban areas with industrial activities. These areas are characterized by the overlap of high levels of pollution with a high population density (over 300 people/100 m × 100 m grid cell) and the built environment (over 7% of the city surface being moderately to very densely built), thus configuring zones with high potential for population exposure.

At the same time, factors that influence pollutant dispersion and accumulation, such as DistHS and BuildH&F, amplify these patterns. The low DistHS values (over 71% of the analyzed area being at a distance of less than 4 km from pollution sources and hot spots in terms of E_PM₁₀_), corroborated with the presence of dense urban structures and vertical development, favor the stagnation and recirculation of PM in the atmosphere, limiting ventilation and dispersion processes. In contrast, the variables associated with attenuation effects, namely NDVI and LE, highlight an important role in reducing the exposure potential. Areas characterized by high NDVI values (67.1% of the OMA has medium and high NDVI values) and higher altitudes are generally associated with conditions more favorable to PM dispersion and with a lower atmospheric load (Figure 8).

The spatial distribution of the potential health impact of PM_10_ exposure in 2025 at the OMA level highlights the predominance of medium- and low-exposure classes. At the annual level, approximately 51.9% of the territory falls into the medium exposure class, followed by 39.7% in the low class and only 8.3% in the high class, suggesting a moderate generalized impact, with limited areas where the risk is more pronounced.

**Figure 9:**
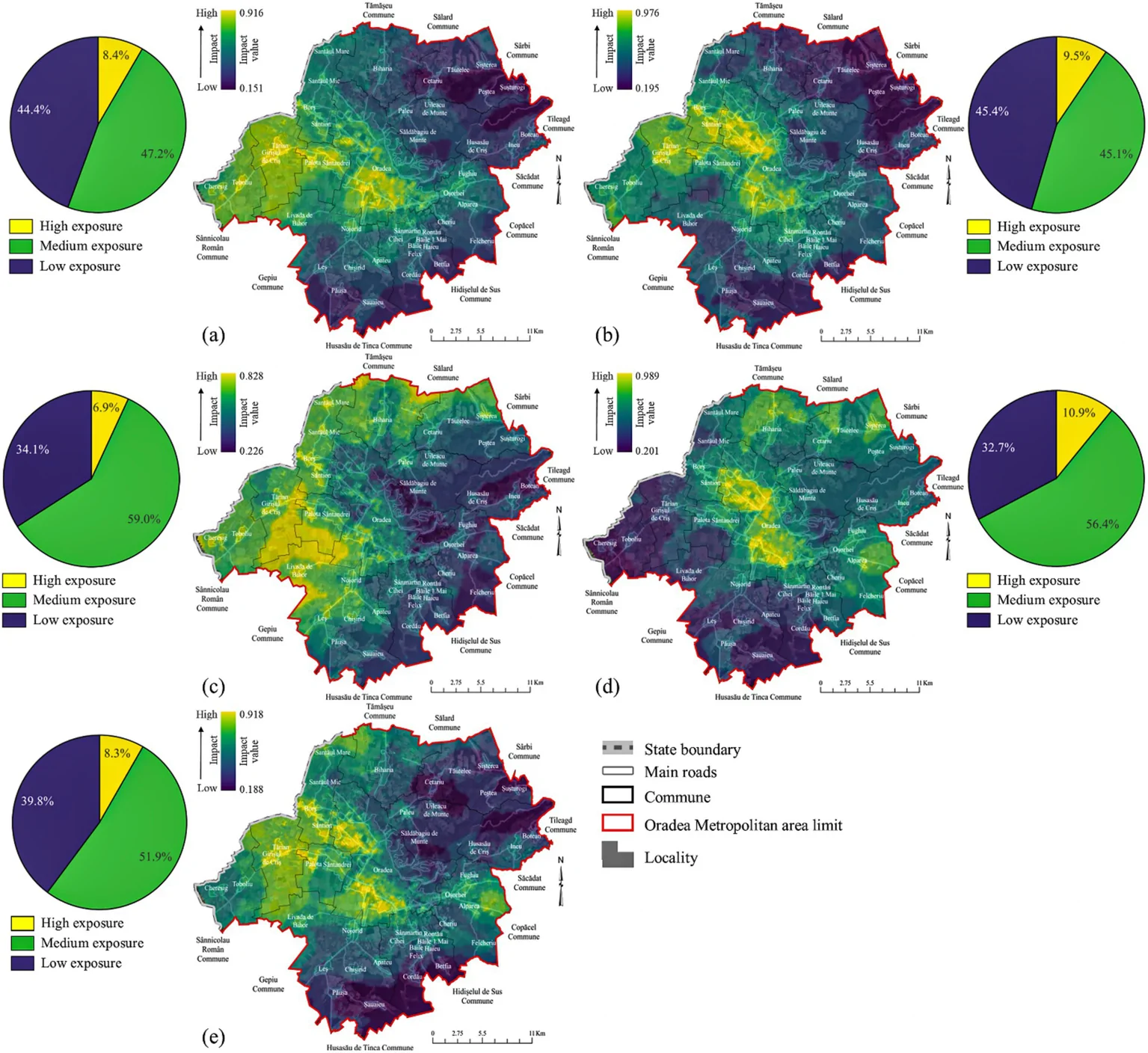
Spatial distribution of the potential health impact of PM_10_ exposure for 2025, derived from a weighted multi-criteria composite index, illustrating seasonal averages **(a)** Winter; **(b)** Spring; **(c)** Summer; **(d)** Autumn and the annual mean **(e)** across the study area.

The seasonal analysis indicates relevant variations in the distribution of these classes. The largest increase in the area with high exposure is recorded in autumn (10.9%), followed by spring (9.5%) and winter (8.4%), while summer shows the lowest share of this class (6.9%). Regarding average exposure, the maximum values occur in the summer (59%) and autumn (56.4%), while the lowest values are in spring (45.1%). Low-exposure classes have the largest spatial extent in spring (45.5%) and winter (44.4%), whereas in autumn (32.7%) and summer (34.1%) their share is lower (Figure 9).

Overall, the results indicate that autumn corresponds to the season with the highest potential health impact, while summer is characterized by a low incidence of high exposure but by the dominance of the medium-exposure class. At the annual level, the distribution is dominated by moderate-risk areas, while both severe- and minimal-impact areas have relatively low shares.

Regarding the spatial distribution of the potential health impact associated with PM_10_ exposure, the northern and southern areas of Oradea Municipality are clearly highlighted as having the highest exposure levels. These zones are characterized by proximity to the main industrial sites and high population density, factors that cumulatively amplify the risk. Also, high exposure values are highlighted in the western sector of the OMA, especially in the communes of Sântandrei, Girișu de Criș and Borș. These areas become more pronounced in summer and winter, suggesting the combined influence of regional pollutant transport, specific meteorological conditions, and the expansion of anthropogenic activities in the city’s vicinity (Figure 9).

The distribution of the population by exposure level shows a clear predominance of medium-exposure zones, with approximately 198,373 inhabitants, representing 70.6% of the OMA’s total population. At the same time, high-exposure zones account for 56,478 inhabitants (20.1%), indicating locations where potential health risks are amplified by elevated pollution levels. In contrast, low-exposure zones include 26,131 inhabitants (9.3%), suggesting a relatively limited extent of areas with favorable air quality.

## Discussion

The relationship between AOD and PM₁₀ concentrations is influenced by numerous factors and is characterized by high variability, often being nonlinear (18, 34). On a seasonal scale, this relationship can even become inverse, given that AOD values tend to peak in the warm season, while PM₁₀ concentrations are usually higher in the cold season (35). This variability is largely determined by atmospheric processes that control the vertical distribution of aerosols. Thus, under conditions characterized by a high Boundary Layer Height (BLH), PM disperses vertically, leading to lower concentrations at ground level, whereas a low BLH favors their accumulation near the surface. BLH is generally higher in the warm season, due to increased atmospheric turbulence (36, 37). In addition, the AOD–PM₁₀ relationship can be significantly influenced by processes in the free atmosphere, introducing an additional layer of variability into the observed correlations. Since AOD integrates the contribution of aerosols over the entire atmospheric column, the presence of high-altitude aerosol layers, generated by long-range transport or by regional and global atmospheric circulation, can lead to a decoupling between AOD and PM_10_ values (38, 39). Under these conditions, PM-laden air masses can indirectly influence population exposure even in the absence of significant local sources, especially in situations characterized by atmospheric stability or thermal inversions (40, 41). Therefore, understanding the influence of upper-tropospheric atmospheric circulation is very important for interpreting the AOD–PM₁₀ relationship, as it can lead to both overestimation of aerosol loading and masking of local contributions, but such data are limited and difficult to quantify and integrate into models.

In this context, the relatively low correlation between AOD and PM₁₀ concentrations observed in this study confirms the well-known limitations of using AOD as a direct proxy for ground-level PM₁₀ (17, 18). It is worth noting that the relatively low coefficient of determination observed for the direct AOD–PM₁₀ relationship in the present study is consistent with previous studies reporting weak-to-moderate or highly variable relationships between satellite-derived AOD and ground-level PM. Such variability has been associated with meteorological conditions, boundary-layer dynamics, aerosol vertical distribution, and local topography (6, 42, 43). Similar uncertainties in satellite-derived AOD have also been reported in Central and Eastern Europe (44), while research conducted in Romania has highlighted that the relationship between satellite-derived AOD and ground-level PM₁₀ varies considerably across monitoring locations (45) and indicates the benefits of integrating satellite information with ground-based observations for PM₁₀ assessment (46). Thus, in accordance with the above, AOD was not used as a standalone predictor in the proposed model. Its integration with MEs improved the representation of PM₁₀ variability, highlighting the important role of meteorological conditions in controlling pollutant dispersion, vertical mixing, and accumulation (8, 14, 15). The reliability of the resulting estimates is further supported by the relatively moderate prediction errors (RMSE = 7.8 μg/m^3^; MAE = 2.9 μg/m^3^) and by the LOOCV results (RMSE = 8.16 μg/m^3^; MAE = 4.36 μg/m^3^), which indicate that the magnitude of the prediction error remains reasonably stable when individual observations are excluded from model calibration. Nevertheless, the weak direct AOD–PM₁₀ relationship represents an important limitation and indicates that the model should not be interpreted as a universal predictive relationship. Rather, it represents a locally calibrated approach intended to improve the spatial representation of PM₁₀ in an urban area characterized by a limited ground-monitoring network.

E_PM₁₀_ values register high levels in certain spatial contexts within the OMA, as a result of a combination of anthropogenic factors, including proximity to industrial areas, traffic intensity and density of the built environment, as well as the continuous process of urban expansion and infrastructure development. Also, agricultural activities in peri-urban areas can be significant sources of PM under certain conditions. Comparing E_PM₁₀_ values with the internationally established thresholds highlights important differences depending on the reference framework used. Thus, in relation to the WHO guidelines (47), which recommend an annual average value of 15 μg/m^3^ and a daily limit of 45 μg/m^3^, the values obtained in this study constantly exceed the annual threshold, the estimated average being approximately 20.5 μg/m^3^, and in the cold season reaching average values of up to 31.7 μg/m^3^ and absolute values of over 41 μg/m^3^. In contrast to European Union standards, which set annual and daily limits of 40 μg/m^3^ and 50 μg/m^3^ (48), E_PM₁₀_ values are generally within the permissible limits. This discrepancy highlights that, although PM₁₀ levels can be considered normatively acceptable, in some situations they exceed the values recommended by international organizations for optimal health protection. Exceeding these thresholds can have significant adverse effects on human health, as confirmed by numerous recent epidemiological studies and meta-analyses (49). Exposure to high concentrations of PM₁₀ is correlated with an increased risk of overall mortality, as well as cardiovascular and respiratory mortality (50–53), and multi-city studies highlight that even moderate increases in concentrations can cause health effects, especially in vulnerable people (children, the older population, people undergoing treatment, with low immunity, etc.) (54).

However, it is important to note that not all PM directly contributes to adverse effects on human health, as their composition is highly heterogeneous. PM₁₀ includes both anthropogenic particles with high toxic potential (e.g., heavy metals, organic compounds, or elemental carbon) and natural or secondary particles such as water droplets, salts, or biogenic aerosols, which may have a low impact on health (55). In addition, atmospheric processes such as hygroscopic growth can increase particle size by absorbing water vapor, thereby influencing measured concentrations without necessarily indicating a proportional increase in toxicity. However, standard monitoring methods do not allow clear differentiation between less harmful fractions and those with high toxic potential, which limits the direct interpretation of PM₁₀ values solely from the perspective of health risks (56, 57).

To more rigorously model the influence of E_PM₁₀_ on population health, an integrated approach was adopted, incorporating SSIs operationalized via a composite exposure index. This approach allows a more faithful representation of spatial patterns of exposure by capturing the interactions between environmental and anthropogenic factors. In line with the specialized literature (7, 9), the results highlight that population exposure is not determined exclusively by PM₁₀ concentrations, but by the complex interaction among pollution, population distribution, built-environment characteristics, and atmospheric dispersion conditions. In this context, the composite index developed in the study enables identification of areas with a high predisposition to exposure, particularly where pollution levels coincide with high population density and urban structures that favor pollutant stagnation. The analysis highlights that approximately 20.1% of the resident population lives in areas highly prone to elevated PM₁₀ levels, primarily in the immediate vicinity of the two industrial platforms in Oradea Municipality. Although these areas are not characterized by highly polluting industrial activities, the results suggest they contribute significantly to PM generation and accumulation. This contribution can be explained by diffuse emissions associated with light industry-specific production processes (e.g., material handling, mechanical and logistical processes), as well as by the intensification of road traffic and related transport and storage activities, which favor particle resuspension and increased PM concentrations at the local level (58, 59). In addition, the numerous construction sites across the urban area are a significant source of PM₁₀, contributing through dust emissions and the resuspension of PM, often with cumulative effects near industrial and high-traffic zones.

Direct comparison with international satellite-based PM studies should account for substantial differences in spatial scale, monitoring network density, modelling strategy, and input data availability. Recent studies have demonstrated the potential of integrating satellite-derived AOD with MEs, land-use and ancillary information for estimating ground-level PM concentrations (60–64). These studies generally reported *R*^2^ values in the range of approximately 0.70–0.80, although such performance was commonly achieved using considerably denser monitoring networks, larger training datasets and more complex machine-learning approaches, including Random Forest, gradient boosting and deep-learning architectures. Consequently, these values are not directly comparable with those obtained from a locally calibrated multiple linear regression model developed from a limited monitoring network. In the present study, despite the availability of only four regulatory monitoring stations, prediction errors remained relatively moderate, and LOOCV yielded comparable error magnitudes. This suggests that although the model explains a moderate proportion of PM₁₀ variability compared with data-intensive machine-learning approaches, its absolute prediction errors remain reasonably constrained for the intended spatial-exposure application. The methodological differences should therefore be considered in relation to the specific objectives, spatial scale, data availability, and monitoring infrastructure of each study. In this context, the present approach provides a complementary framework for satellite-based PM₁₀ estimation in medium-sized urban areas, where the availability and spatial density of ground-based monitoring observations may be more limited than in large regional- or national-scale studies.

## Conclusion

The results of this study indicate that estimating PM₁₀ concentrations from AOD satellite data, integrated with MEs, can be done with satisfactory accuracy, offering a viable alternative given the limitations of ground-based monitoring networks. Moreover, combining E_PM₁₀_ values with SSIs in a composite index has proven effective for identifying areas with high pollution levels and quantifying the exposed population, enabling an integrated assessment of risk from both spatial and demographic perspectives.

Although the relationship between AOD and PM₁₀ is not always linear and is influenced by various atmospheric factors, the results show that AOD can effectively highlight areas with high pollution levels under certain conditions. In the case of OMA, these areas are located predominantly in the western sector and in the vicinity of the municipality’s two industrial platforms. This suggests that although industrial activities are not highly polluting (light industry being a major contributor), they remain significant sources of PM₁₀. At the same time, the contribution of road traffic and other anthropogenic activities in dense urban areas appears less significant to the overall distribution of AOD and E_PM₁₀_, as these areas, especially the city center, do not show substantial increases in these values.

The key results of the study show that the real risk associated with PM₁₀ exposure is not determined solely by concentration levels, but by their interaction with population density and built-environment characteristics, which amplifies the impact in certain urban settings. The integrated approach indicates that approximately 20.1% of the resident population lives in high-exposure zones, while only 9.3% reside in low-exposure zones, highlighting an uneven distribution of risk concentrated in specific sectors of the OMA. The results have direct implications for urban planning and air quality management, highlighting the need to implement differentiated measures based on spatial exposure patterns. In particular, areas identified as vulnerable require priority interventions, both by reducing emissions and by optimizing the structure of the built environment and increasing urban green spaces. The proposed approach can also support the substantiation of public health policies aimed at reducing population exposure to pollution. Importantly, the methodological framework has potential for application to other urban environments, particularly medium-sized cities characterized by limited ground-based air-quality monitoring networks. Its transferability relies on the use of widely available satellite, meteorological, and spatial datasets, allowing the general workflow to be reproduced in different geographical contexts. However, local calibration and validation remain necessary to account for differences in emission sources, meteorological conditions, urban morphology, and aerosol characteristics. Therefore, the proposed framework should be regarded as a transferable methodological approach rather than a universally parameterized model.

## Limitations and future works

The relationship between AOD and PM₁₀ concentrations is moderate, and MEs and SSIs do not fully explain the variability at ground level, particularly because AOD is a vertically integrated measure of aerosols, which can introduce uncertainties in estimating surface concentrations. In addition, satellite-derived AOD retrievals are affected by cloud contamination, which reduces the number of valid observations and limits the temporal continuity of the analysis. Consequently, although a longer study period would have been desirable, only the year 2025 provided a sufficiently continuous and consistent dataset of satellite observations and corresponding ground-based measurements suitable for model development. Furthermore, the presence of only four official air quality monitoring stations within the OMA limits the spatial representation of PM₁₀ and MEs, thereby constraining the robustness of model calibration. Seasonal meteorological variability and changes in atmospheric mixing conditions also influence the relationship between AOD and ground-level PM₁₀, contributing additional uncertainty to the estimates. Finally, because the proposed model was developed and calibrated exclusively for the OMA, its direct transferability to other urban environments should be approached with caution without local recalibration.

The model used, based on multiple linear regression, also represents a simplified approach to the complex processes governing PM₁₀ concentrations. Future research will therefore focus on developing a more robust database by extending the analysis over multiple years, integrating data from additional urban areas and, where available, denser monitoring networks, auxiliary low-cost sensors, and higher-resolution satellite products. As larger multi-year satellite and synchronized ground-based datasets become available, these improvements will provide a stronger basis for implementing and evaluating non-linear machine-learning approaches, particularly ensemble methods such as Random Forest and XGBoost, which may better capture complex interactions between AOD, MEs, and ground-level PM₁₀ concentrations. The increased spatial density of ground observations could also support the development of continuous spatial uncertainty maps using geostatistical approaches, including Kriging-based methods, thereby enabling more comprehensive assessment of prediction uncertainty and its propagation into composite population exposure estimates. Finally, the proposed framework could be extended to other major atmospheric pollutants (PM₂.₅, CO, NO₂, and SO₂), enabling a more comprehensive assessment of urban air quality and population exposure.

## Data Availability

The raw data supporting the conclusions of this article will be made available by the authors, without undue reservation.

## Glossary

AOD: Aerosol Optical Depth
AP: atmospheric pressure
BLH: Boundary Layer Height
BuildD: density of built-up areas
BuildH&F: the height and function of buildings
DistHS: distance from hot spots with high E_PM₁₀_ values
E_PM₁₀_: estimated value of Particulate Matter ≤10 μm
LE: land elevation
MAIAC: Multi-Angle Implementation of Atmospheric Correction
MEs: meteorological elements
MODIS: Moderate Resolution Imaging Spectroradiometer
NDVI: Normalized Difference Vegetation Index
OMA: Oradea Metropolitan Area
PM: Particulate Matter
PM_10_: Particulate Matter ≤10 μm
Pop: population density
RH: relative humidity
SR: solar radiation
SSIs: socio-spatial indicators
T: temperature
WHO: World Health Organisation
WS: wind speed
LOOCV: Leave-One-Out Cross-Validation
RMSE: Root Mean Square Error
MAE: Mean Absolute Error
MBE: Mean Bias Error
MAPE: Mean Absolute Percentage Error

## References

[1] Dockery DW. Health effects of particulate air pollution. Ann Epidemiol. (2009) 19:257–63. doi: 10.1016/j.annepidem.2009.01.018, 19344865 PMC3367838

[2] Lim SS Vos T Flaxman AD Danaei G Shibuya K Adair-Rohani H . A comparative risk assessment of burden of disease and injury attributable to 67 risk factors and risk factor clusters in 21 regions, 1990–2010: a systematic analysis for the global burden of disease study 2010. Lancet. (1990) 380:2224–60. doi: 10.1016/s0140-6736(12)61766-8, 23245609 PMC4156511

[3] Samoli E Stafoggia M Rodopoulou S Ostro B Declercq C Alessandrini E . Associations between fine and coarse particles and mortality in Mediterranean cities: results from the MED-PARTICLES project. Environ Health Perspect. (2013) 121:932–8. doi: 10.1289/ehp.1206124, 23687008 PMC3734494

[4] IHME, Global Burden of Disease (2025) With major Processing by Our World in Data. “Outdoor ozone Pollution – IHME” IHME, Global Burden of Disease “Global Burden of Disease - Risk Factors - Deaths”

[5] WHO (2024) Ambient (Outdoor). Geneva, Switzerland: Air Pollution. Available online at: https://www.who.int/news-room/fact-sheets/detail/ambient-(outdoor)-air-quality-and-health (Accessed April 3, 2026).

[6] Stirnberg R Cermak J Andersen H. An analysis of factors influencing the relationship between satellite-derived aerosol optical depth and ground-level PM10. Remote Sens. (2018) 10:1353. doi: 10.3390/rs10091353

[7] Di Q Amini H Shi L Kloog I Silvern R Kelly J . An ensemble-based model of PM2.5 concentration across the contiguous United States with high spatiotemporal resolution. Environ Int. (2019) 130:104909. doi: 10.1016/j.envint.2019.104909 31272018 PMC7063579

[8] Zhang X Chu Y Wang Y Zhang K. Predicting daily PM2.5 concentrations in Texas using high-resolution satellite aerosol optical depth. Sci Total Environ. (2018) 631-632:56–70. doi: 10.1016/j.scitotenv.2018.03.019, 29728001

[9] Beloconi A Chrysoulakis N Lyapustin A Utzinger J Vounatsou P. Bayesian geostatistical modelling of PM10 and PM2.5 surface level concentrations in Europe using high-resolution satellite-derived products. Environ Int. (2018) 121:57–70. doi: 10.1016/j.envint.2018.08.041, 30179765 PMC6295977

[10] Putaud J-P Van Dingenen R Alastuey A Bauer H Birmili W Cyrys J . A European aerosol phenomenology—3: physical and chemical characteristics of particulate matter from 60 rural, urban, and kerbside sites across Europe. Atmos Environ. (2010) 44:1308–20. doi: 10.1016/j.atmosenv.2009.12.011

[11] Sameh S Zaki A Elsaka B Beshr AAA Shehata AG. Air pollution mapping and monitoring using sentinel-5P data and Google earth engine. Egypt J Remote Sens Space Sci. (2026) 29:158–78. doi: 10.1016/j.ejrs.2026.01.008

[12] Sameh S Zarzoura F El-Mewafi M. Spatio-temporal analysis mapping of air quality monitoring in Cairo using Sentinel-5 satellite data and Google earth engine. Mansoura Eng J. (2023) 49:1–18. doi: 10.58491/2735-4202.3122

[13] Aljarbouai SJS Zarzoura F El-Mewafi M Sameh S. The impact of atmospheric pollutants on thermal conditions in Riyadh, Saudi Arabia based on Google earth engine and geographically weighted regression model (GWR). Innov Infrastruct Solut. (2026) 11:269. doi: 10.1007/s41062-026-02650-w

[14] Gupta P Christopher SA. Particulate matter air quality assessment using integrated surface, satellite, and meteorological products: multiple regression approach. J Geophys Res Atmos. (2009) 114:D14205. doi: 10.1029/2008JD011496, 29143083

[15] Kloog I Koutrakis P Coull BA Lee HJ Schwartz J. Assessing temporally and spatially resolved PM2.5 exposures for epidemiological studies using satellite aerosol optical depth measurements. Atmosph Environ. (2011) 45:6267–75. doi: 10.1016/j.atmosenv.2011.08.066

[16] van Donkelaar A Martin RV Brauer M Kahn R Levy R Verduzco C . Global estimates of ambient fine particulate matter concentrations from satellite-based aerosol optical depth: development and application. Environ Health Perspect. (2010) 118:847–55. doi: 10.1289/ehp.0901623, 20519161 PMC2898863

[17] Wang J Christopher SA. Intercomparison between satellite-derived aerosol optical thickness and PM2.5 mass: implications for air quality studies. Geophys Res Lett. (2003) 30:2095 doi: 10.1029/2003GL018174

[18] Chudnovsky A Tang C Lyapustin A Wang Y Schwartz J Koutrakis P. A critical assessment of high-resolution aerosol optical depth retrievals for fine particulate matter predictions. Atmos Chem Phys. (2013) 13:10907–17. doi: 10.5194/acp-13-10907-2013

[19] Buya S Usanavasin S Gokon H Karnjana J. An estimation of daily PM2.5 concentration in Thailand using satellite data at 1-kilometer resolution. Sustainability. (2023) 15:10024. doi: 10.3390/su151310024

[20] Pavolonis M Sieglaff J. GOES-R Advanced Baseline Imager (ABI) Algorithm Theoretical Basis Document for Volcanic Ash (Detection and Height). Madison, WI: University of Wisconsin (2010).

[21] Unik M Sitanggang IS Syaufina L Jaya INS. PM2.5 estimation using machine learning models and satellite data: a literature review. Int J Adv Comput Sci Appl. (2023) 14:538. doi: 10.14569/IJACSA.2023.0140538

[22] El Kihel M Caciora T Grava C Țarcă R-C Saafadi L Ouallali A. Integrating multi-source remote sensing and ensemble machine learning to quantify the surface urban heat island in Oradea municipality, Romania. Urban Clim. (2026) 67:102872. doi: 10.1016/j.uclim.2026.102872

[23] Caciora T. El Kihel M. Blaga L. Herman G. V. Costea M. (2026). Assessment of Spatio-temporal Dynamics of SUHI and UHI Using MODIS Land Surface Temperature and Ground-based Observations in a Mid-sized European city. City and Environment Interactions (under Review).

[24] Croitoru A-E Banc Ș Scripcă A-S Rus A-V. Climatic suitability for outdoor tourism in Romania’s big cities. Atmosphere (Basel). (2024) 15:996. doi: 10.3390/atmos15080996

[25] Dumiter AF. Clima și Topoclimatele orașului Oradea [Climate and Topoclimates of the city of Oradea]. Oradea: Editura Universității din Oradea (2007).

[26] Dumitrescu A Bojariu R Birsan M-V Marin L Manea A. Recent climatic changes in Romania from observational data (1961–2013). Theor Appl Climatol. (2015) 122:111–9. doi: 10.1007/s00704-014-1290-0

[27] Lyapustin A Wang Y Korkin S Huang D. MODIS collection 6 MAIAC algorithm. Atmos Meas Tech Discuss. (2018):5741–5765. doi: 10.5194/amt-11-5741-2018

[28] Lyapustin AI Wang Y Laszlo I Hilker T Hall F Sellers PJ . Multi-angle implementation of atmospheric correction for MODIS (MAIAC): 3. Atmospheric correction. Remote Sens Environ. (2012) 127:385–93. doi: 10.1016/j.rse.2012.09.002

[29] Lyapustin A Wang Y. MCD19A2 MODIS/Terra+ Aqua Land Aerosol Optical DEPTH Daily L2G Global 1 km SIN gr id V006 [Data Set]. Sioux Falls, South Dakota: NASA EOSDIS Land Processes DAAC (2018).

[30] Pesaresi M Schiavina M Politis P Freire S Krasnodębska K Uhl JH . Advances on the global human settlement layer by joint assessment of earth observation and population survey data. Int J Digit Earth. (2024) 17:2390454. doi: 10.1080/17538947.2024.2390454

[31] Abdul Shakor AS Pahrol MA Mazeli MI. Effects of population weighting on PM10 concentration estimation. J Environ Public Health. (2020) 2020:1–11. doi: 10.1155/2020/1561823, 32351580 PMC7174967

[32] Hart JE Yanosky JD Puett RC Ryan L Dockery DW Smith TJ . Spatial modeling of PM10 and NO2 in the continental United States, 1985-2000. Environ Health Perspect. (2009) 117:1690–6. doi: 10.1289/ehp.0900840, 20049118 PMC2801201

[33] Gulliver J de Hoogh K Fecht D Vienneau D Briggs D. Comparative assessment of GIS-based methods and metrics for estimating long-term exposures to air pollution. Atmosph Environ. (2011) 45:7072–80. doi: 10.1016/j.atmosenv.2011.09.042

[34] Barnaba F Putaud JP Gruening C Dell’Acqua A Dos Santos S. Annual cycle in co-located in situ, total-column, and height-resolved aerosol observations in the Po Valley (Italy): implications for ground-level particulate matter mass concentration estimation from remote sensing. J Geophys Res Atmos. (2010) 115:1–22. doi: 10.1029/2009JD013002

[35] Arvani B Pierce RB Lyapustin AI Wang Y Ghermandi G Teggi S. Seasonal monitoring and estimation of regional aerosol distribution over Po valley, northern Italy, using a high-resolution MAIAC product. Atmos Environ. (2016) 141:106–21. doi: 10.1016/j.atmosenv.2016.06.037

[36] Boyouk N Léon JF Delbarre H Podvin T Deroo C. Impact of the mixing boundary layer on the relationship between PM2.5 and aerosol optical thickness. Atmos Environ. (2010) 44:271–7. doi: 10.1016/j.atmosenv.2009.06.053

[37] Van Donkelaar A Martin RV Park RJ. Estimating ground-level PM2.5 using aerosol optical depth determined from satellite remote sensing. J Geophys Res Atmos. (2006) 111:1–10. doi: 10.1029/2005JD006996

[38] Hoff RM Christopher SA. Remote sensing of particulate pollution from space: have we reached the promised land? J Air Waste Manag Assoc. (2009) 59:645–75. doi: 10.3155/1047-3289.59.6.645, 19603734

[39] Schaap M Apituley A Timmermans RMA Koelemeijer RBA de Leeuw G. Exploring the relation between aerosol optical depth and PM2.5 at different spatial scales. Atmos Chem Phys. (2009) 9:909–25. doi: 10.5194/acp-9-909-2009

[40] Boys BL Martin RV van Donkelaar A MacDonell RJ Hsu NC Cooper MJ . Fifteen-year global time series of satellite-derived fine particulate matter. Environ Sci Technol. (2014) 48:11109–18. doi: 10.1021/es502113p, 25184953

[41] Paciorek CJ Liu Y. Limitations of remotely sensed aerosol as a spatial proxy for fine particulate matter. Environ Health Perspect. (2009) 117:904–9. doi: 10.1289/ehp.0800360, 19590681 PMC2702404

[42] Benas N Beloconi A Chrysoulakis N. Estimation of urban PM10 concentration, based on MODIS and MERIS/AATSR synergistic observations. Atmos Environ (Oxford, England: 1994). (2013) 79:448–54. doi: 10.1016/j.atmosenv.2013.07.012

[43] Emili E Popp C Wunderle S Zebisch M Petitta M. Mapping particulate matter in alpine regions with satellite and ground-based measurements: an exploratory study for data assimilation. Atmos Environ (Oxford, England: 1994). (2011) 45:4344–53. doi: 10.1016/j.atmosenv.2011.05.051

[44] Ajtai N Mereuta A Stefanie H Radovici A Botezan C Zawadzka-Manko O . SEVIRI aerosol optical depth validation using AERONET and intercomparison with MODIS in central and eastern Europe. Remote Sens. (2021) 13:844. doi: 10.3390/rs13050844

[45] Barladeanu R Stefan S Radulescu R. Correlation between the particulate matter (PM10) mass concentrations and aerosol optical depth in Bucharest, Romania. Rom Rep Phys. (2012) 64:1085–96.

[46] Dumitrache RC Iriza A Maco BA Barbu CD Hirtl M Mantovani S . Study on the influence of ground and satellite observations on the numerical air-quality for PM10 over Romanian territory. Atmos Environ. (2016) 143:278–89. doi: 10.1016/j.atmosenv.2016.08.063

[47] World Health Organization. WHO Global air Quality Guidelines: Particulate Matter (PM2.5 and PM10), ozone, Nitrogen Dioxide, sulfur Dioxide and carbon Monoxide. Geneva: WHO (2021). 34662007

[48] European Commission (2008) Directive 2008/50/EC of the European Parliament and of the Council of 21 May 2008 on Ambient Air Quality and Cleaner air for Europe Strasbourg, France: Official Journal of the European Union.

[49] Ilieș A Caciora T Ilieș G Hassan TH Berdenov Z Ilieș DC . An integrated risk assessment model for indoor air quality. PLoS One. (2025) 20:e0336594. doi: 10.1371/journal.pone.0336594 41370267 PMC12694796

[50] Caciora T Ilieș DC Costea M Blaga L Berdenov Z Ilieș A . Microclimate assessment in a 19th-century heritage building from Romania. Indoor Air. (2024) 2024:2989136. doi: 10.1155/2024/2989136

[51] Ilies A Caciora T Marcu F Berdenov Z Ilies G Safarov B . Analysis of the interior microclimate in art nouveau heritage buildings for the protection of exhibits and human health. Int J Environ Res Public Health. (2022) 19:16599. doi: 10.3390/ijerph192416599, 36554480 PMC9779619

[52] Karimi B. Shokrinezhad B., & others. (2024). Short-term exposure to particulate matter (PM10) and mortality: a systematic review and meta-analysis. Environ Res, 240:117456. doi: 10.1016/j.envres.2023.117456 37866540

[53] Guo C Zhang Z Lau AKH Lin CQ Chuang YC Chan J . Effect of long-term exposure to fine particulate matter on mortality and morbidity: a systematic review and meta-analysis. Sci Total Environ. (2023) 832:155021. doi: 10.1016/j.scitotenv.2022.155021

[54] Orellano P Quaranta N Reynoso J Balbi B Vasquez J. Effect of outdoor air pollution on asthma exacerbations in children and adults: systematic review and multilevel meta-analysis. PLoS One. (2020) 15:e0222382. doi: 10.1371/journal.pone.0222382 PMC5358780 28319180

[55] Pope CA Dockery DW. Health effects of fine particulate air pollution: lines that connect. J Air Waste Manag Assoc. (2006) 56:709–42. doi: 10.1080/10473289.2006.10464485, 16805397

[56] Harrison RM Jones AM Lawrence RG. A pragmatic mass closure model for airborne particulate matter at urban background and roadside sites. Atmospheric Environ. (2003) 37:4927–33. doi: 10.1016/j.atmosenv.2003.08.025

[57] Kelly FJ Fussell JC. Size, source and chemical composition as determinants of toxicity attributable to ambient particulate matter. Atmos Environ (Oxford, England: 1994). (2012) 60:504–26. doi: 10.1016/j.atmosenv.2012.06.039

[58] Amato F Cassee FR Denier van der Gon HAC Gehrig R Gustafsson M Hafner W . Urban air quality: the challenge of traffic non-exhaust emissions. J Hazard Mater. (2014) 275:31–6. doi: 10.1016/j.jhazmat.2014.04.053, 24837462

[59] Pant P Harrison RM. Estimation of the contribution of road traffic emissions to particulate matter concentrations from field measurements: a review. Atmospheric Environment (Oxford, England: 1994). (2013) 77:78–97. doi: 10.1016/j.atmosenv.2013.04.028

[60] Chen G Wang Y Li S Cao W Ren H Knibbs LD . Spatiotemporal patterns of PM10 concentrations over China during 2005-2016: a satellite-based estimation using the random forests approach. Environ Pollut. (2018) 242:605–13. doi: 10.1016/j.envpol.2018.07.012, 30014938

[61] Stirnberg R Cermak J Fuchs J Andersen H. Mapping and understanding patterns of air quality using satellite data and machine learning. J Geophys Res Atmos. (2020) 125:e2019JD03138 doi: 10.1029/2019jd031380

[62] Chen B Song Z Shi B Li M. An interpretable deep forest model for estimating hourly PM10 concentration in China using Himawari-8 data. Atmos Environ. (2022) 268:118827. doi: 10.1016/j.atmosenv.2021.118827

[63] Park S Im J Kim J Kim S-M. Geostationary satellite-derived ground-level particulate matter concentrations using real-time machine learning in Northeast Asia. Environ Pollut. (2022) 306:119425. doi: 10.1016/j.envpol.2022.119425, 35537556

[64] Das A Sahu M. Leveraging satellite data for predicting PM10 concentration with machine learning models: a study in the plains of North Bengal, India. Aerosol Air Qual Res. (2024) 24:240066. doi: 10.4209/aaqr.240066

